# Prevalence of *Borrelia burgdorferi* in Ixodidae Tick around Asia: A Systematic Review and Meta-Analysis

**DOI:** 10.3390/pathogens11020143

**Published:** 2022-01-24

**Authors:** Zhenhua Ji, Miaomiao Jian, Peng Yue, Wenjing Cao, Xin Xu, Yu Zhang, Yingyi Pan, Jiaru Yang, Jingjing Chen, Meixiao Liu, Yuxin Fan, Xuan Su, Shiyuan Wen, Jing Kong, Bingxue Li, Yan Dong, Guozhong Zhou, Aihua Liu, Fukai Bao

**Affiliations:** 1 The Institute for Tropical Medicine, Faculty of Basic Medical Sciences, Kunming Medical University, Kunming 650500, China; jizhenhua5@126.com (Z.J.); m18227112867@163.com (M.J.); 13778452230@163.com (P.Y.); cwj8166@163.com (W.C.); trustafew@163.com (X.X.); 18228024528@163.com (Y.Z.); winniep1424@163.com (Y.P.); yjrakm@126.com (J.Y.); a18975094706@163.com (J.C.); liumeixiao1@163.com (M.L.); fanyuxin1998@163.com (Y.F.); suxuanss12345@163.com (X.S.); shiyuanwen267@126.com (S.W.); kongjing1@kmmu.edu.cn (J.K.); libingxue1@kmmu.edu.cn (B.L.); m18875040118@163.com (Y.D.); zhouguozhong@kmmu.edu.cn (G.Z.); 2 Third Affiliated Hospital of Kunming Medical University (Yunnan Cancer Hospital), Kunming 650100, China; 3 Yunnan Province Key Laboratory of Children’s Major Diseases Research, The Affiliated Children Hospital, Kunming Medical University, Kunming 650030, China

**Keywords:** Lyme disease, *Borrelia burgdorferi*, tick, Ixodes, meta-analysis

## Abstract

Lyme disease (LD) is a common arthropod-borne inflammatory disorder prevalent in the northern hemisphere. LD is caused by a spirochete named *Borrelia burgdorferi* s.l., which is transmitted to humans by ticks. Climate, environment, and other factors affect land use; recreational-behavior changes affect human contact with infected ticks. Studies in Europe and North America have looked at these aspects, but studies in Asia have not. We searched databases to identify all relevant abstracts published until March 2021. A meta-analysis was undertaken using the standard methods and procedures established by the Cochrane Collaboration. Ninety-one articles were included in our meta-analysis. The literature search identified data from nine countries (China, Japan, Malaysia, Mongolia, Pakistan, Russia Siberia region, South Korea, Thailand and Turkey). Furthermore, 53,003 ticks from six genera (*Amblyomma*, *Dermacentor*, *Haemaphysalis*, *Hyalomma*, *Ixodes* and *Rhipicephalus*) were inspected for infection with *B. burgdorferi.* The pooled prevalence was 11.1% (95% CI = 8.3–14.2%). Among the nine countries, China had the most studies (56) and Malaysia had the highest infection rate (46.2%). Most infected ticks were from the genera *Ixodes* and *Haemaphysalis*. Ticks of the genus *Ixodes* had the highest infection rate (16.9%). Obvious heterogeneity was noted in our meta-analysis. We analyzed the heterogeneity with regard to countries, genera, time points, and detection methods. This study suggests that *Ixodes*, *Haemaphysalis* and *Dermacentor* may be the most common tike of *B. burgdorferi*-positive in Asia. The highest proportion of ticks infected by *B. burgdorferi* were from the genus *Ixodes.* This meta-analysis is the first attempt to explain the *B. burgdorferi* infection of hard-body ticks in Asia. The infection rate for each country and infection rate of different tick genera were analyzed: there were large differences between them. The literature is concentrates mainly on East Asia, and data are limited. Our study can provide a reference for a more comprehensive and in-depth investigation of ticks in Asia infected by *B. burgdorferi* spirochetes.

## 1. Introduction

Lyme disease (LD) is a tick-borne inflammatory disease caused by infection with *Borrelia burgdorferi* sensu lato (*B. burgdorferi* s.l.) complex. LD is of public-health importance in moderate-climate regions of the northern hemisphere, such as North America, Europe, North Africa, and Northern Asia. As landscapes have altered, the number of reported cases have revealed obvious differences in many regions such as *Ixodes ricinus*, *Ixodes persulcatus*, etc.

The clinical symptoms of LD can be divided into three stages. Erythema migrans (the most common clinical manifestation) is a typical sign of early acute infection [1]. It is an expanding skin redness that usually develops at the site of a tick bite. Often, several weeks to months after the tick bite, followed by early dissemination and development, *B. burgdorferi* s.l. can spread to other tissues and organs, and untreated infections can progress to neurologic abnormalities or heart dysfunction [2,3]. Usually, late LD manifests as arthritis or acrodermatitis chronica atrophicans, and is associated with spirochete invasion of joints [4,5,6]. A fatal outcome from LD is extremely rare.

There are several *B. burgdorferi* s.l. genospecies, and not all strains/genotypes cause LD in humans. *B. burgdorferi sensu stricto, Borrelia afzelii*, *Borrelia garinii,* and *Borrelia bavariensis* are considered to be of pathogenic relevance to humans. Despite cases of LB caused by *Borrelia valaisiana*, *Borrelia lusitaniae*, and *Borrelia bissettiae* have been described, their pathogenic ability has been questioned and their description is occasional. *Borrelia mayonii* has been recently incorporated in the Americas [7,8]. Globally, three genospecies of *B. burgdorferi* are principally pathogenic to humans. *Borrelia burgdorferi* sensu stricto (hereafter referred to as *B. burgdorferi*) is distributed mainly in the Americas. *Borrelia afzelii* and *Borrelia garinii* infections are predominant in LD cases in Europe. *B. garinii* is the primary cause of LD in Asia [9]. Other species, such as *Borrelia bissettii*, *Borrelia lusitaniae*, and *Borrelia valaisiana*, are also considered to cause human LD, but the prevalence of infections is low and so they are not considered to be major pathogens [10,11]. Interestingly, the genotype of pathogens seems to be the main factor causing the diversity of clinical symptoms of LD. For example, *B. afzelii* most frequently leads to skin lesions, *B. burgdorferi* is especially arthritogenic, and *B. garinii* is linked to neuroborreliosis [12,13].

Different *B. burgdorferi* s.l. genospecies are transmitted by different genera of ticks, and some ticks can be infected with multiple genospecies of *B. burgdorferi* s.l. The main vectors transmitting LD-associated spirochetes to humans are *Ixodes ricinus* in Europe, *Ixodes persulcatus* in Asia, *Ixodes scapularis* in eastern North America, and *Ixode pacificus* in Western North America [14]. These vectors have four life stages (egg, larva, nymph, and adult). In the last three feeding stages, ticks require a blood meal from a variety of mammals, birds, and lizards [13]. The lifecycle of spirochetes in nature is dependent upon horizontal transmission between an infected tick and vertebrate host. Typically, tick larvae acquire spirochetes from infectious hosts via a blood meal. Spirochetes are carried in the midgut of ticks, and transmitted to susceptible host populations through injection of tick saliva during tick feeding. *B. burgdorferi* replicates in the mammalian dermis, and then disseminates to distant cutaneous sites and other organs, including joints [15]. 

The risk to humans of infection with *Borrelia* depends on outdoor recreational activity, on the density of tick populations, and on the infection of the ticks with *Borrelia* [16]. *I.persulcatus* is the prevalent vector in the southern forest zone on the Asian side of Eurasis, from the western border of Russia to its far eastern frontier bordering China, Korea, and Japan. However, on the Western sade of Eurasia, most European countries and North Africa harbor *I. ricinus*. *I. ricinus*, which is the most common tick species that bites humans in the study area and in most European countries. The jury is still out on the main spirochete transmission tick in Asia [17]. Identification of the genotypes of LD-associated spirochetes, geographic range, and understanding of the distribution of their vectors have essential epidemiologic and clinical importance. Meta-analysis of the prevalence and distribution of *B. burgdorferi* s.l. genospecies in ticks in Europe has been undertaken but, in Asia, such analyses are lacking [16,18,19]. A number of field studies have already pointed to increases in average densities and activities of questing ticks in parts of Europe with long-documented *I. ricinus* populations. Studies have identified a strong negative correlation between tick density and altitude, which is related to local climatic conditions [20].

In this study, we aimed to systematically analyze the existing literature on the prevalence of *Borrelia burgdorferi* in ticks in Asia. Tick prevalence was assessed by tick species, sampling area, and detection methods. The work made crude estimates of tick spirochete infection rates in Asia. It is hoped that this study can provide a more comprehensive and in-depth investigation of ticks infected with Borrelia burgdorferi in Asia.

## 2. Results

### 2.1. Search Results and Study Selection

**Figure 1:**
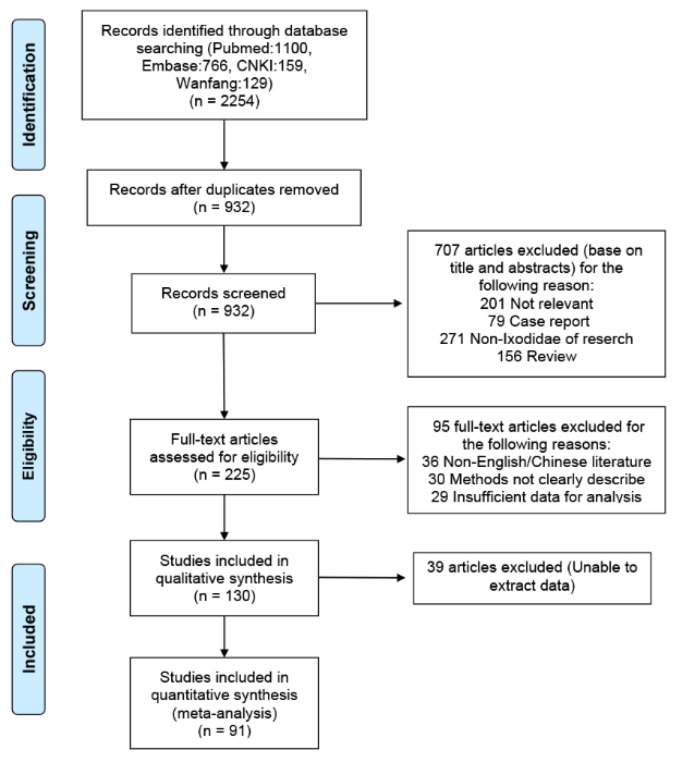
Flowchart of our study.

A total of 2254 titles with abstracts were screened, 225 full-text articles were reviewed, and 91 articles were included in this study (Supplementary Materials 3 and 4). Initially, we identified 2254 records through four databases. After elimination of duplicates, 932 records remained. We screened the titles and abstracts and excluded 592 irrelevant records. We scrutinized the full text of the remaining 225 papers for eligibility, of which 130 were excluded. Through screening, we identified data from 91 articles that were suitable for our meta-analysis (56 in English and 35 in Chinese), which reported 91 studies from countries and 160 studies on species. Details of the article-screening procedure and reasons for exclusion are summarized in Figure 1.

### 2.2. Study Characteristics

**Figure 2:**
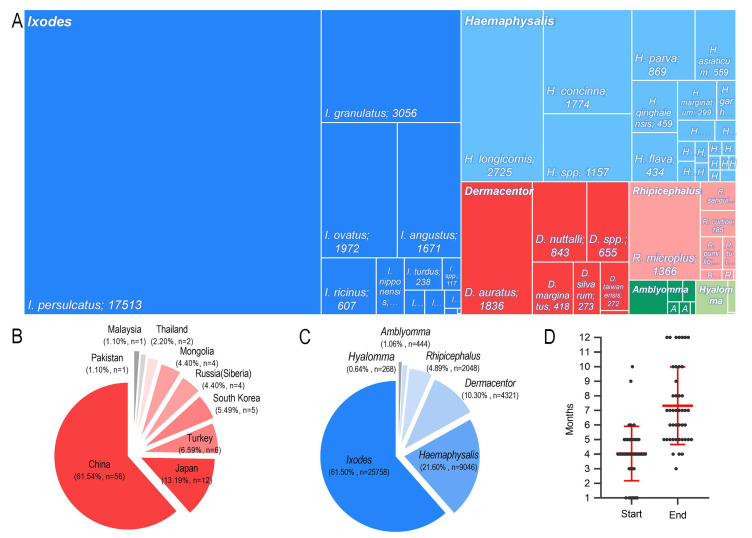
Study characteristics. (**A**) Rectangular dendrogram of Ixodid species. (**B**) Proportion of studies from each country. (**C**) Proportion of each genus. (**D**) Tick capture time.

Reports identified by the database search were first assessed for eligibility by their titles and abstracts, followed by an in-depth analysis for relevant data regarding *B. burgdorferi* prevalence in the Ixodid tick family Ixodidae. Of the 91 included studies, 61 (67.00%) had a “low” risk, 30 (33.00%) had a “moderate” risk, and 0 (0.00%) had a “high” risk of bias. The studies were cross-sectional, and 56 studies were reported from China, 12 from Japan, six from Turkey, five from South Korea, four from Mongolia, four from Russia (Siberia), two from Thailand, one from Malaysia, and one from Pakistan. Nine countries reported the rates of ticks infected with *B. burgdorferi* in Asia, with China and Japan accounting for 74.73% of the total number of studies. Therefore, most data were from East Asia. A total of 53,003 ticks were involved in this meta-analysis, and the number of *B. burgdorferi*-positive ticks was 7777. Among these ticks, most were from the genera *Ixodes* and *Haemaphysalis*, followed by *Rhipicephalus*, *Amblyomma*, and *Hyalomma*. The collected literature was published between 1990 and 2010. Most of the ticks checked were caught between April and July, and most of them were detected by polymerase chain reaction (PCR). The detailed characteristics of each study are provided in Figure 2.

### 2.3. Pooling and Heterogeneity of Selected Studies

**Figure 3:**
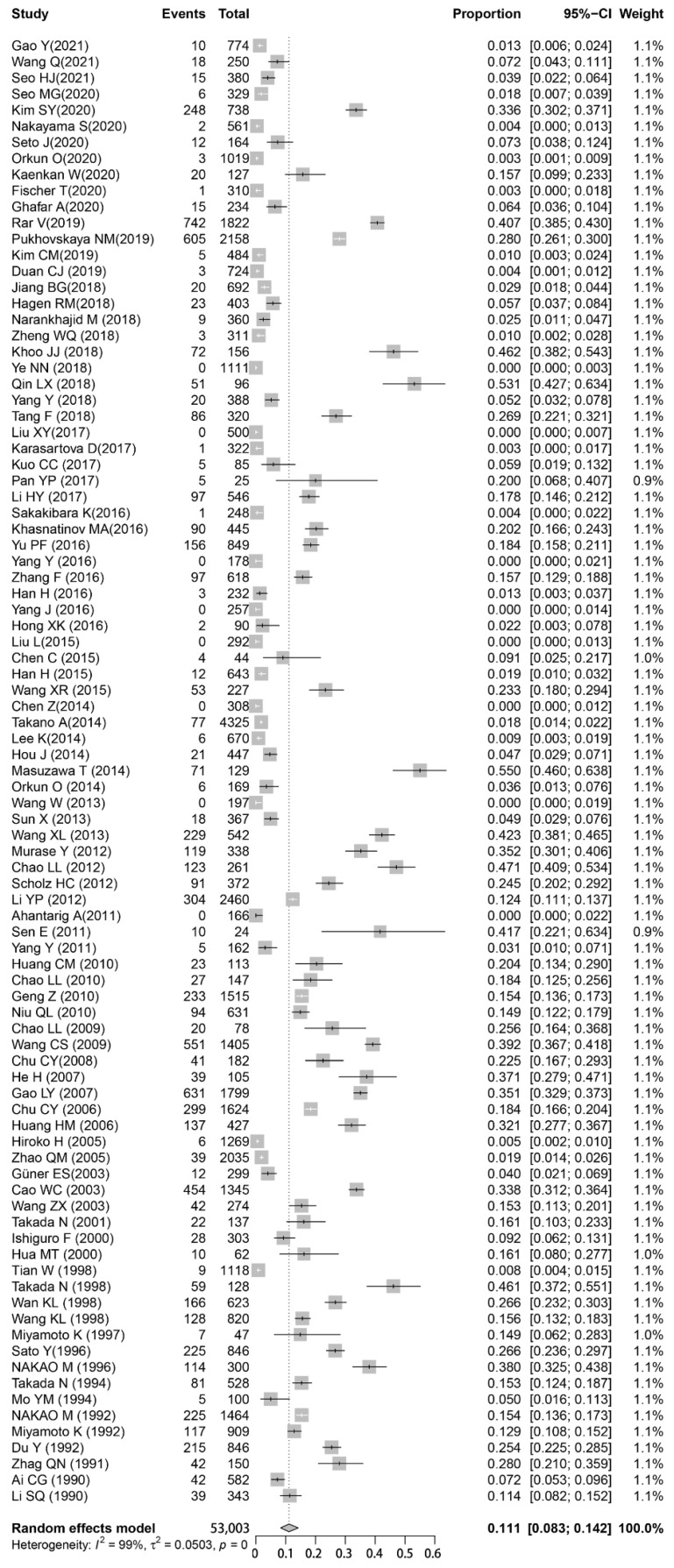
Forest plot showing the prevalence of *B. burgdorferi* s.l. in Ixodidae. Events: Number of *Borrelia*-positive ticks; Total: Number of ticks detected. Please refer to Supplementary Material 3, 4 for details.

The pooled prevalence was calculated based on a random-effects model, with all studies being included in our meta-analysis. The pooled prevalence of infection by *B. burgdorferi* was 11.1% (95% confidence interval (CI) = 8.3–14.2%), and significant heterogeneity was found regarding the pooled prevalence (I^2^ = 0.99; *p* < 0.001). The rate of infection by *B. burgdorferi* in ticks among the included studies varied between 0% and 55% (Figure 3).

### 2.4. Country

**Figure 4:**
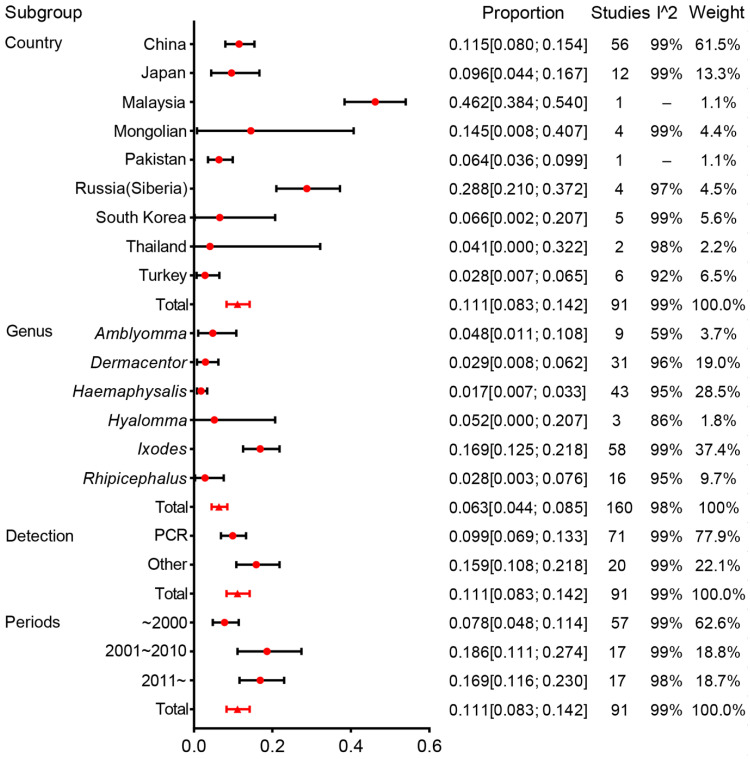
Forest plot of the prevalence of *B. burgdorferi* s.l. in Ixodidae by subgroup. Red circles denote the infection rate estimated by random effects meta-analysis and whisker bars denote 95%CI. Subgroups according to country, genus, detection, and periods. Results (bottom line, *n* = 95) are shown for all included studies. Please refer to Supplementary Material 5 for details.

The estimates of prevalence for different countries and genera, and heterogeneities are presented in Figure 4. Estimates of infection rates for different subgroups and heterogeneities are presented in Table 1. Pooled infection rates for each subgroup were calculated using a random-effects model because of the observed high heterogeneity among studies within subgroups.

In the survey on Asian prevalence, 91 studies were conducted from nine countries, and 53,003 ticks were checked. The prevalence results were 11.5% (95%CI, 8.0–15.4%) for China, 9.6% (4.4–16.7%) for Japan, 46.2% (38.4–54.0%) for Malaysia, 14.5% (8.0–40.7%) for Mongolia, 6.4% (3.6–9.9%) for Pakistan, 28.8% (21.0–37.2%) for Russia (Siberia), 6.6% (0.2–20.7%) for South Korea, 4.1% (0.0–32.2%) for Thailand, and 2.8% (0.7–6.5%) for Turkey.

**Figure 5:**
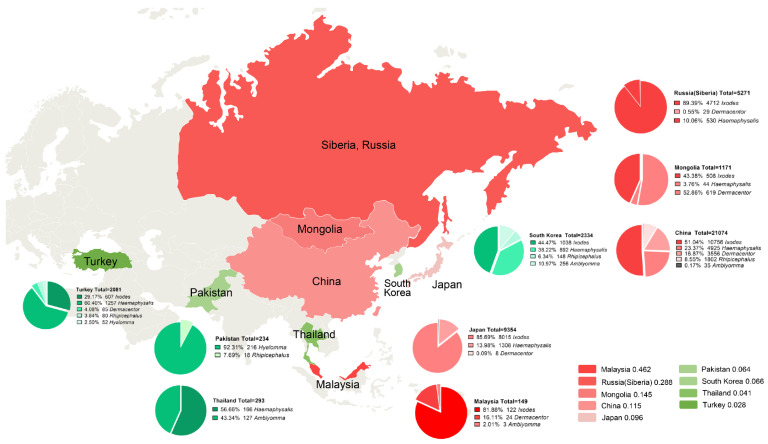
Distribution of included studies. Red: Top five countries with tick infection; Green: Tick infection rate after four countries; Pie chart: Percentage of tick species per country.

Hence, big differences in the species and genera of Ixodidae in different countries were documented. *Ixodes* and *Haemaphysalis* were the main genera in China, Japan, South Korea, Turkey, and Siberia. *Dermacentor* was the genus with the largest proportion in Mongolia. *Rhipicephalus* was distributed in China, South Korea, Turkey, and Pakistan, but the *Rhipicephalus* tested number was relatively small. *Amblyomma* was distributed mainly in Thailand, South Korea, and Malaysia. *Hyalomma* was found only in Turkey and Pakistan. In all studies, China accounted for 61.5% of the weight and contributed significantly to the results of the study. Only one study was done in Malaysia, so we had doubts about the high rate of infection documented in that study (Figure 5).

### 2.5. Genus

**Figure 6:**
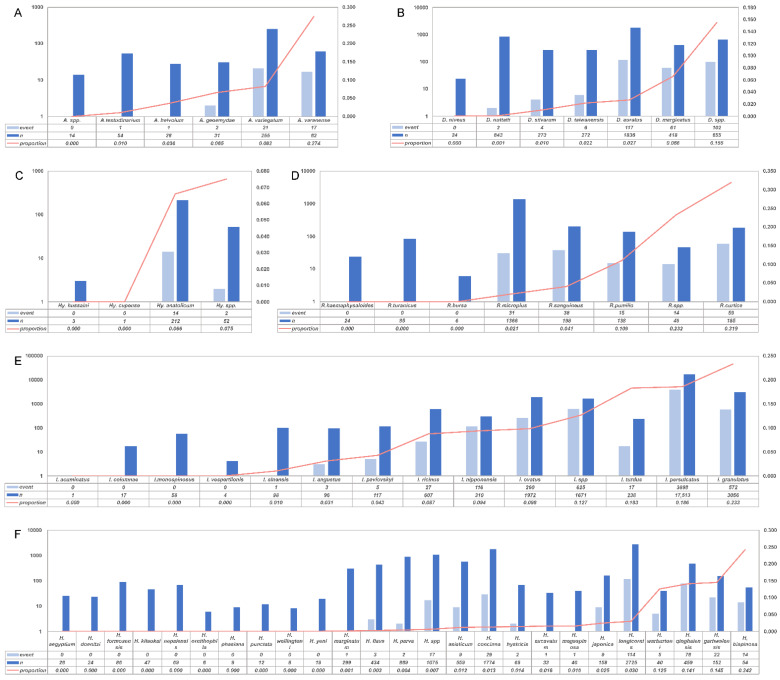
Infection rate according to species. (**A**): *Amblyomma* infection rates; (**B**): *Dermacentor* infection rates; (**C**): *Hyalomma* infection rates; (**D**): *Rhipicephalus* infection rates; (**E**): *Ixodes* infection rates; (**F**): *Haemaphysalis* infection rates; event: ticks that test positive for spirochetes were shown as light blue; *n*: total number of ticks detected were shown as blue; proportion: *Borrelia*-positive rate.

In our analyses, 41,885 ticks were identified to genera, and the prevalence at the genus level could be calculated. *Ixodes* had an infection rate of 16.9% (95% CI, 12.5–21.8%), whereas it was 1.7% (0.7–3.3%) for *Haemaphysalis*, 2.9% (0.8–6.2%) for *Dermacentor*, 2.8% (0.3–7.6%) for *Rhipicephalus*, 4.8% (1.1–10.8%) for *Amblyomma*, and 5.2% (0.0–20.7%) for *Hyalomma*. Of tick genera infected with *B. burgdorferi* (which explained 36.1% of the heterogeneity), an infection rate of 16.9% from the *Ixodes* genus was higher than that of other genera. Among the genus were classified further, and *I. persulcatus* and *I. granulatus* were the most numerous and had a higher infection rate. The tick species with the most frequently identified in *Amblyomma*, *Dermacentor*, *Rhipicephalus*, and *Hyalomma* were *Amblyomma variegatum*, *Dermacentor auratus*, *Rhipicephalus microplus*, and *Hyalomma anatolicum*, and the infection rate was 8.2%, 2.7%, 2.1%, and 6.6%, respectively (Figure 6).

The meta-regression analysis revealed that the country, genus, period of publication, and detection methods were the source of heterogeneity (Table 1).

### 2.6. Publication Bias

Egger’s linear regression test was undertaken and a funnel plot was constructed to examine the publication bias (Supplementary Information). They showed that the studies had a symmetrical distribution. Egger’s test (*t* = −0.181, *p* = 0.857) did not show a significant value.

## 3. Discussion

LD occurs most frequently in the Northern Hemisphere, where some ticks of the Ixodidae family are present. Each year, ~300,000 people in the USA and ≤85,000 people in Europe are infected with *B. burgdorferi* s.l. and suffer LD [21,22]. Although the true incidence of LD in Asian populations is not known, its distribution appears to be widening.

Ticks are ectoparasites that carry multiple pathogens. They transmit these pathogens to humans and animals. Persistent and relapsing infection as well as long-term sequelae caused by tick-borne pathogens worsen human health further. As the infection rate is very high, animal husbandry is a global economic burden. [23,24].

One of the most notable functions of ticks is that they serve as vectors of LD. In LD, there is a dynamic interplay between spirochetes, vectors, and reservoir hosts. The spirochetes involved in LD hold a wide range of reservoir hosts, so clarifying the distribution of infected ticks in Asia could help for estimating the prevalence of *B. burgdorferi* s.l. and improve the prevention and control of LD. 

Ticks transmit a wide range of pathogens into humans and animals. In North America, *I. scapularis* and *I. pacificus* have been shown to be vectors of the major LD-causing spirochete *B. burgdorferi* s.l. *I. ricinus* and *I. persulcatus* have been confirmed experimentally to be the carriers of LD-causing spirochetes in Eurasia [25,26]. Due to the genetic diversity of ticks, the relative abundance of certain pathogens is quite different across different tick genera [27]. None of the eight tick species from three genera (one species from the genus *Amblyomma*, five from *Dermacentor*, and two from *Haemaphysalis*) evaluated to date have been unequivocally and experimentally confirmed to be vectors of *B. burgdorferi* s.l. spirochetes [25,28,29]. The host specialization and/or vector compatibility of LD spirochetes may affect the distribution of spirochetes of different genospecies. 

The genetic structure and pathogen composition of different tick genera are affected mainly by ecologic and geographic factors. For example, *H. longicornis* is a widely distributed tick species indigenous to eastern Asia, whereas *Hyalomma asiaticum* prefers to live in desert or semi-desert environments, *I. ricinus* is distributed widely at high altitudes [19,30,31]. Within an endemic area, the risk of infection by *B. burgdorferi* s.l. in humans is determined by the local abundance and infection rate of vector ticks, and by human behavior that affects the likelihood of being bitten. Research on tick genera infected with spirochetes helps public-health agencies make strategies to prevent LD.

With a total area of land and population, China is the largest country in Asia. China has a total area of ~9.6 million km^2^, which is almost the size of Europe. Due to influencing factors such as the size of geographic area and number of reports on infected ticks, our included data were concentrated mainly in East Asia (especially China). With tick activity, LD shows relatively constant regional characteristics and seasonal peaks. The habitat types of ticks and local microclimate determine the abundance of infected ticks, which affects LD prevalence. Numerous tick species are expanding beyond their historical distribution range and invading new regions, and the increase in the number of human cases of tick-borne disease is concomitant with such an expansion [32,33]. We showed that the typical habitats of uninfected/infected ticks were woodlands and grasslands in regions with mild climates, which tallies with the geographic range of LD transmission. These habitats provide sufficient humidity for the development and survival of ticks and vertebrate hosts. We demonstrated that ticks usually become active from spring to late summer, which is consistent with the peak incidence of LD in humans. LD in humans is also correlated with meteorological conditions that influence tick feeding and human behavior, such as temperature, humidity, and rainfall [19,34,35]. Ticks usually feed on blood meals in the summer, which is the same time that recreation by humans increases. In areas with a high incidence of ticks, the annual average temperature is stable at 6.85–16.85 °C, and spring vegetation is lush [19].

Understanding the distribution of ticks spcies can help in the prevention and diagnosis of LD. Equally important, differences among genospecies of *B. burgdorferi* s.l. are thought to cause variability in the clinical symptoms of LD in different geographic areas. We analyzed the geographic and genus distribution of ticks infected with *B. burgdorferi* s.l. Furthermore, our study contains data primarily on China, which are not generalizable to other large areas. *I. persulcatus* and *I. granulatus* were the most numerous and had a higher infection rate. Consistent with previous research, *I.persulcatus* is the prevalent vector in the southern forest zone on the Asian side of Eurasis, from the western border of Russia to its far eastern frontier bordering China, Korea, and Japan. In Southeast Asia and West Asia, tick infection rates are low and data collection is low, and more studies should be added. In conclusion, this meta-analysis is the first attempt to explain the B. burgdorferi infection of hard-body ticks in Asia. Our study can provide a reference for more comprehensive and in-depth investigations of ticks in Asia infected by B. burgdorferi spirochetes.

## 4. Materials and Methods

### 4.1. Search Strategy

In this meta-analysis, two independent investigators searched PubMed, Excerpta Medica Database (Embase), the China National Knowledge Infrastructure, and Wanfang databases to identify all relevant abstracts published until March 2021. The key search terms were “*Ixodes*” OR “Ixodidae” OR “Tick” AND “*Borrelia*” AND “Asia”. Titles and abstracts of articles retrieved from the literature search were screened independently by two investigators. The full text of potentially eligible studies were obtained and assessed further for final inclusion. A third investigator analyzed any inconsistent results to resolve discrepancies.

### 4.2. Literature Search and Data Extraction

Studies were considered eligible only if they: (i) were carried out within Asia; (ii) were a surveillance report or cross-sectional study, neither experimental studies nor review articles; (iii) the study object was Ixodidae; (iv) were written in English or Chinese.

The exclusion criteria were: (i) incomplete data; (ii) the study was a review, case report, or comment to editors (lacking primary data); (iii) the study was a repeated publication.

After training, two individuals reviewed the abstracts independently and identified articles for detailed assessment. In case of disagreement, the two parties discussed and resolved the issue or referred it to a third researcher for a final decision. Then, they extracted data from each included study and entered the results into a database. Data on the first author, year of publication, country, sample-collection sites, screening test used, sample size, and number of infections were extracted.

### 4.3. Quality of Evidence and Risk of Bias

The methodological quality of included studies was evaluated using the tool developed by Hoy and colleagues [36]. A score of 1 (“yes”) or 0 (“no”) was assigned for each item. Scores were summed across items to generate an overall quality score that ranged from 0 to 10 (Supplementary Information). Then, studies were classified as having a low (>8), moderate [6,7,8], or high (≤5) risk of bias. Four investigators independently assessed the methodological quality of one-quarter of included studies each, and all assessments were reviewed independently by a fifth investigator, with disagreements being resolved through consensus.

### 4.4. Data Analyses

Extracted data were entered into Excel 2016 within Office (Microsoft, Redmond, WA, USA). The meta-analysis was conducted using the “meta” package in R 3.1.3 (R Project for Statistical Computing, Vienna, Austria) to estimate the prevalence of *B. burgdorferi* in ticks from Ixodidae. We used valid classification lists of tick genera [37]. Our study was carried out in accordance with Preferred Reporting Items for Systematic Reviews and Meta-analyses (PRISMA) guidelines [38]. The PRISMA checklist (Supplementary Information) was used as the basis for inclusion of relevant information.

A forest plot and funnel plot were generated to judge the overall effect size and ascertain if publication bias was present. Heterogeneity among studies was assessed using Cochran’s Q test (reported as *p*-values), which is quantified by I^2^ values. If there was evidence of heterogeneity (I^2^ > 50%), infection rates were combined using a random-effects model; otherwise, infection rates were combined using a fixed-effects model. Unadjusted prevalence was recalculated on the basis of the information of crude numerators and denominators provided by individual studies. The pooled prevalence and its 95%CI of *B. burgdorferi* in Ixodidae were calculated with the Freeman–Tukey double arcsine transformation [39]. Publication bias was assessed using Egger’s test and funnel plots [40].

The effects of heterogeneity on seroprevalence estimates were examined by subgroup and meta-regression analyses. Such analyses were undertaken on different variables: country, tick genus, species-detection method, and time the research was done.

## 5. Conclusions

This meta-analysis is the first attempt to explain the *B. burgdorferi* infection of Ixodid ticks in Asia. The infection rate for each country and infection rate of different tick genera were analyzed: there were large differences between them. The literature is concentrates mainly on East Asia, and data are limited. Our study can provide a reference for a more comprehensive and in-depth investigations of ticks in Asia infected by *B. burgdorferi* spirochetes.

## Figures and Tables

**Table 1:** Influence analysis in meta-analysis.

Factors Related to Infection Rate		No. of Study Included	No. of Total Tick Examined	Pooled Infection Rate (95%CI)	Metaregression Analysis			
					*p* Value	Tau^2	I^2	R^2
Country		91	53,003	0.111(0.083–0.142)	<0.0001	0.0426	98.96%	15.35%
	China	56	30,585	0.115(0.080–0.154)	<0.0001	-	-	-
	Japan	12	10,878	0.096(0.044–0.167)	<0.0001	-	-	-
	Malaysia	1	156	0.462(0.384–0.540)	0.0004	-	-	-
	Mongolian	4	1171	0.145(0.008–0.407)	0.0002	-	-	-
	Pakistan	1	234	0.064(0.036–0.099)	0.2207			
	Russia (Siberia)	4	5271	0.288(0.210–0.372)	<0.0001			
	South Korea	5	2334	0.066(0.002–0.207)	0.0052			
	Thailand	2	293	0.041(0.000–0.322)	0.1729			
	Turkey	6	2081	0.028(0.007–0.065)	0.0236	-	-	-
Genus		160	41,885	0.063(0.044–0.085)	<0.0001	0.0445	97.82%	29.85%
	*Amblyomma*	9	444	0.048(0.011–0.108)	0.0732	-	-	-
	*Dermacentor*	31	4321	0.029(0.008–0.062)	<0.0001	-	-	-
	*Haemaphysalis*	43	9046	0.017(0.007–0.033)	<0.0001	-	-	-
	*Hyalomma*	3	268	0.052(0.000–0.207)	0.0807	-	-	-
	*Ixodes*	58	25,758	0.169(0.125–0.218)	<0.0001	-	-	-
	*Rhipicephalus*	16	2048	0.028(0.003–0.076)	0.0040	-	-	-
Periods		91	53,003	0.111(0.083–0.142)	<0.0001	0.0482	99.08%	4.30%
	~2000	57	30,449	0.078(0.048–0.114)	<0.0001	-	-	-
	2001~2010	17	13,385	0.186(0.111–0.274)	<0.0001	-	-	-
	2011~	17	9169	0.169(0.116–0.230)	<0.0001	-	-	-
Detection		91	53,003	0.111(0.083–0.142)	<0.0001	0.0467	99.07%	7.16%
	PCR	71	39,528	0.099(0.069–0.133)	<0.0001	-	-	-
	Other	20	13,475	0.159(0.108–0.218)	<0.0001	-	-	-
Country + Periods		91	53,003	0.111(0.083–0.142)	<0.0001	0.0379	98.81%	24.68%
Country + Detection		91	53,003	0.111(0.083–0.142)	<0.0001	0.0378	98.82%	24.85%
Country + Periods+ Detection		91	53,003	0.111(0.083–0.142)	<0.0001	0.0369	98.76%	26.76%

* *p* < 0.05, covariate effects were statistically significant; tau^2, estimated amount of residual heterogeneity; I^2, residual heterogeneity/unaccounted variability; R^2, amount of heterogeneity accounted for.

## Data Availability

The data that support the findings of this survey are available from the corresponding author upon reasonable request.

## Supplementary Materials

The following are available online at https://www.mdpi.com/article/10.3390/pathogens11020143/s1, Supplementary Material 1: Assessment of risk of bias; Supplementary Material 2: PRISMA checklist; Supplementary Material 3: Meta-analysis included articles; Supplementary Material 4: Study characteristic; Supplementary Material 5: Asia prevalence of B. burgdorferi in Ixodidae by subgroup(country); Supplementary Material 6: Asia prevalence of *B. burgdorferi* in Ixodidae by subgroup(genus).
