# Supplementary material for: Prevalence of Borrelia burgdorferi in Ixodidae Tick around Asia: A Systematic Review and Meta-Analysis
Source: Pathogens. 2022 Jan 24;11(2):143. doi: 10.3390/pathogens11020143 (PMC8879681; doi:10.3390/pathogens11020143)

Supplementary material for：

Prevalence of *Borrelia burgdorferi* in Ixodidae tick around Asia: a systematic review and meta-analysis

Content

Material 1: Assessment of risk of bias · 1

Material 2: PRISMA checklist · 4

Material 3: Meta-analysis included articles · 6

Material 4: Study characteristic· 12

Material 5: Asia prevalence of *B. burgdorferi* in Ixodidae by subgroup(country) · 20

Material 6: Asia prevalence of *B. burgdorferi* in Ixodidae by subgroup(genus) · 30

Supplementary Material 1: Assessment of risk of bias

**Items**
**Was the study's target population a close representation of the population in relation to relevant variables?**<br>*0. No or Unclear*<br>*1. Clear description of infection diagnosis: Polymerase Chain Reaction (PCR), immunofluorescent antibody assay (IFA), Direct Immunofluorescent Assay (DFA), Spirochete Culture, and so on*
**Was the sampling frame a true or close representation of the target population?**<br>*0. No or Unclear*<br>*1. Yes*
**Was some form of random selection used to select the sample, OR was a census undertaken?**<br>*0. No or Unclear*<br>*1. Yes*
**Was the likelihood of nonresponse bias minimal?**<br>*0. No or Unclear*<br>*1. Yes*
**Were data collected directly from the subjects (as opposed to a proxy)?**<br>*0. No: proxy, databases, medical reports only*<br>*1. Directly from the subjects*
**Was an acceptable case definition of positive infection used in the study?**<br>*0. Unclear*<br>*1. Laboratory-based*
**Was the study method that diagnosed Positive *B.burgdorferi* infection shown to have validity and reliability?**<br>*0. No description of the method*<br>*1. Yes: Polymerase Chain Reaction (PCR), immunofluorescent antibody assay (IFA), Direct Immunofluorescent Assay (DFA), Spirochete Culture, and so on*
**Was the same mode of data collection used for all subjects?**<br>*0. No or Unclear*<br>*1. Yes*
**Was the length of the shortest prevalence period for the parameter of interest appropriate?**<br>*0. No or Unclear*<br>*1. Yes*
**Were the numerator(s) and denominator(s) for the parameter of interest appropriate and clearly reported?**<br>*0. No or Unclear*<br>*1. Yes*

Study	Authors	Score	Low risk	Moderate risk	High risk
1	Gao Y (2021)	10	+	–	–
2	Seo HJ (2021)	8	–	+	–
3	Wang Q (2021)	10	+	–	–
4	Seo MG (2020)	8	–	+	
5	Kim SY (2020)	9	+	–	–
6	Nakayama S (2020)	8	–	+	–
7	Seto J (2020)	10	+	–	–
8	Orkun Ö (2020)	9	+	–	–
9	Kaenkan W (2020)	9	+	–	–
10	Fischer T (2020)	10	+	–	–
11	Ghafar A (2020)	9	+	–	–
12	Rar V (2019)	9	+	–	–
13	Pukhovskaya NM (2019)	8	–	+	–
14	Kim CM (2019)	10	+	–	–
15	Duan CJ (2019)	9	+	–	–
16	Jiang BG (2018)	10	+	–	–
17	Hagen RM (2018)	9	+	–	–
18	Narankhajid M (2018)	8	–	+	–
19	Zheng WQ (2018)	8	–	+	–
20	Khoo JJ (2018)	8	–	+	–
21	Ye NN (2018)	9	+	–	–
22	Qin LX (2018)	7	–	+	–
23	Yang Y (2018)	9	+	–	–
24	Tang F (2018)	9	+	–	–
25	Liu XY (2017)	10	+	–	–
26	Karasartova D (2017)	10	+	–	–
27	Kuo CC (2017)	7	–	+	–
28	Pan YP (2017)	8	–	+	–
29	Li HY (2017)	10	+	–	–
30	Sakakibara K (2016)	9	+	–	–
31	Khasnatinov MA (2016)	9	+	–	–
32	Yu PF (2016)	9	+	–	–
33	Yang Y (2016)	8	–	+	–
34	Zang F (2016)	10	+	–	–
35	Han H (2016)	9	+	–	–
36	Yang J (2016)	9	+	–	–
37	Hong XK (2016)	9	+	–	–
38	Liu L (2015)	9	+		
39	Chen C (2015)	8	–	+	–
40	Han H (2015)	10	+	–	–
41	Wang XR (2015)	9	+	–	–
42	Chen Z (2014)	10	+	–	–
43	Takano A (2014)	10	+	–	–
44	Lee K (2014)	10	+	–	–
45	Hou J (2014)	8	–	+	–

46	Masuzawa T (2014)	9	+	–	–
47	Orkun O (2014)	8	–	+	–
48	Wang W (2013)	9	+	–	–
49	Sun X (2013)	9	+	–	–
50	Wang XL (2013)	8	–	+	–
51	Murase Y (2012)	9	+	–	–
52	Chao LL (2012)	8	–	+	–
53	Scholz HC (2012)	9	+	–	–
54	Li YP (2012)	10	+	–	–
55	Ahantarig A (2011)	10	+	–	–
56	Sen E (2011)	8	–	+	–
57	Yang Y (2011)	9	+	–	–
58	Huang CM (2010)	8	–	+	–
59	Chao LL (2010)	8	–	+	–
60	Geng Z (2010)	8	–	+	–
61	Niu QL (2010)	10	+	–	–
62	Chao LL (2009)	7	–	+	–
63	Wang CS (2009)	8	–	+	–
64	Chu CY (2008)	9	+	–	–
65	He H (2007)	9	+	–	–
66	Gao LY (2007)	9	+	–	–
67	Chu CY (2006)	10	+	–	–
68	Huang HM (2006)	9	+	–	–
69	Hiroko H (2005)	9	+	–	–
70	Zhao QM (2005)	10	+	–	–
71	Güner ES (2003)	9	+	–	–
72	Cao WC (2003)	10	+	–	–
73	Wang ZX (2003)	7	–	+	–
74	Takada N (2001)	9	+	–	–
75	Ishiguro F (2000)	8	–	+	–
76	Hua MT (2000)	9	+	–	–
77	Tian W (1998)	10	+	–	–
78	Takada N (1998)	9	+	–	–
79	Wan KL (1998)	8	–	+	–
80	Seo MG (2020)	8	–	+	–
81	Miyamoto K (1997)	9	+	–	–
82	Sato Y (1996)	10	+	–	–
83	NAKAO M (1992)	8	–	+	–
84	Takada N (1994)	9	+	–	–
85	Mo YM (1994)	7	–	+	–
86	NAKAO M (1992)	9	+	–	–
87	Miyamoto K (1992)	7	–	+	–
88	Du Y (1992)	9	+	–	–
89	Zhag QN (1991)	8		+	–
90	Ai CX (1990)	9	+	–	–
91	Li SQ (1990)	9	+	–	–

Supplementary Material 2: PRISMA checklist

Section/topic	#	Checklist item	Reported on page #
**TITLE**			
Title	1	Identify the report as a systematic review, meta-analysis, or both.	**Title**
**ABSTRACT**			
Structured summary	2	Provide a structured summary including, as applicable: background; objectives; data sources; study eligibility criteria, participants, and interventions; study appraisal and synthesis methods; results; limitations; conclusions and implications of key findings; systematic review registration number.	**Abstract**
**INTRODUCTION**			
Rationale	3	Describe the rationale for the review in the context of what is already known.	**Introduction, paragraph 1-3**
Objectives	4	Provide an explicit statement of questions being addressed with reference to participants, interventions, comparisons, outcomes, and study design (PICOS).	**Introduction, paragraph 4**
**METHODS**			
Protocol and registration	5	Indicate if a review protocol exists, if and where it can be accessed (e.g., Web address), and, if available, provide registration information including registration number.	**Methods, paragraph 1-8**
Eligibility criteria	6	Specify study characteristics (e.g., PICOS, length of follow-up) and report characteristics (e.g., years considered, language, publication status) used as criteria for eligibility, giving rationale.	**Methods, paragraph 2**
Information sources	7	Describe all information sources (e.g., databases with dates of coverage, contact with study authors to identify additional studies) in the search and date last searched.	**Methods, paragraph 1**
Search	8	Present full electronic search strategy for at least one database, including any limits used, such that it could be repeated.	**Methods, paragraph 1**
Study selection	9	State the process for selecting studies (i.e., screening, eligibility, included in systematic review, and, if applicable, included in the meta-analysis).	**Methods, paragraph 2**
Data collection process	10	Describe method of data extraction from reports (e.g., piloted forms, independently, in duplicate) and any processes for obtaining and confirming data from investigators.	**Methods, paragraph 2-3**
Data items	11	List and define all variables for which data were sought (e.g., PICOS, funding sources) and any assumptions and simplifications made.	**Methods, paragraph 3**
Risk of bias in individual studies	12	Describe methods used for assessing risk of bias of individual studies (including specification of whether this was done at the study or outcome level), and how this information is to be used in any data synthesis.	**Methods, paragraph 4**
Summary measures	13	State the principal summary measures (e.g., risk ratio, difference in means).	**Methods, paragraph 4-6**
Synthesis of results	14	Describe the methods of handling data and combining results of studies, if done, including measures of consistency (e.g., I^2) for each meta-analysis.	**Methods, paragraph 4-6**

Risk of bias across studies	15	Specify any assessment of risk of bias that may affect the cumulative evidence (e.g., publication bias, selective reporting within studies).	**Methods, paragraph 4**
Additional analyses	16	Describe methods of additional analyses (e.g., sensitivity or subgroup analyses, meta-regression), if done, indicating which were pre-specified.	**Methods, paragraph 4**
**RESULTS**			
Study selection	17	Give numbers of studies screened, assessed for eligibility, and included in the review, with reasons for exclusions at each stage, ideally with a flow diagram.	**Results, paragraph 1 Figure1**
Study characteristics	18	For each study, present characteristics for which data were extracted (e.g., study size, PICOS, follow-up period) and provide the citations.	**Results, paragraph 2 Table 1**
Risk of bias within studies	19	Present data on risk of bias of each study and, if available, any outcome level assessment (see item 12).	**Results, paragraph 2**
Results of individual studies	20	For all outcomes considered (benefits or harms), present, for each study: (a) simple summary data for each intervention group (b) effect estimates and confidence intervals, ideally with a forest plot.	**Results, paragraph 3 Figure 2**
Synthesis of results	21	Present results of each meta-analysis done, including confidence intervals and measures of consistency.	**Results, paragraph 2-4 Table1**
Risk of bias across studies	22	Present results of any assessment of risk of bias across studies (see Item 15).	**Results, paragraph 7 Figure 1-3 Table 1**
Additional analysis	23	Give results of additional analyses, if done (e.g., sensitivity or subgroup analyses, meta-regression [see Item 16]).	**Supplementary**
**DISCUSSION**			
Summary of evidence	24	Summarize the main findings including the strength of evidence for each main outcome; consider their relevance to key groups (e.g., healthcare providers, users, and policy makers).	**Discussion, paragraph 1-6**
Limitations	25	Discuss limitations at study and outcome level (e.g., risk of bias), and at review-level (e.g., incomplete retrieval of identified research, reporting bias).	**Discussion, paragraph 6**
Conclusions	26	Provide a general interpretation of the results in the context of other evidence, and implications for future research.	**Discussion, paragraph 6**
**FUNDING**			
Funding	27	Describe sources of funding for the systematic review and other support (e.g., supply of data); role of funders for the systematic review.	**Acknowledgments**

Supplementary Material 3: Meta-analysis included articles

[1] Gao Y, Lv XL, Han SZ, et al. First detection of *Borrelia miyamotoi* infections in ticks and humans from the northeast of Inner Mongolia, China. 2021. Acta Trop. 2021, 217:105857.
[2] Seo HJ, Noh J, Kim HC, et al. Molecular Detection and Phylogenetic Analysis of *Anaplasma* and *Borrelia* Species in Ticks Collected from Migratory Birds at Heuksan, Hong, and Nan Islands, Republic of Korea. Vector Borne Zoonotic Dis. 2021, 21(1):20-31.
[3] Wang Q, Pan YS, Jiang BG, et al. Prevalence of Multiple Tick-Borne Pathogens in Various Tick Vectors in Northeastern China. Vector Borne Zoonotic Dis. 2021;21(3):162-171.
[4] Seo MG, Kwon OD, Kwak D. Molecular Identification of Borrelia afzelii from Ticks Parasitizing Domestic and Wild Animals in South Korea. Microorganisms. 2020;8(5):649.
[5] Kim SY, Kim TK, Kim TY, et al. Geographical Distribution of *Borrelia burgdorferi* sensu lato in Ticks Collected from Wild Rodents in the Republic of Korea. *Pathogens*. 2020;9(11):866.
[6] Nakayama S, Kobayashi T, Nakamura A, et al. Detection of Borrelia DNA in Tick Species Collected from Vegetation and Wild Animals in Fukuoka, Japan. Jpn J Infect Dis. 2020, 73(1):61-64.
[7] Seto J, Tanaka S, Kawabata H, et al. Detection of tick-borne pathogens in ticks from dogs and cats in Yamagata Prefecture, Japan, 2018. Jpn J Infect Dis. 2021, 74(2):122-128.
[8] Orkun Ö, Çakmak A, Nalbantoğlu S, et al. Turkey tick news: A molecular investigation into the presence of tick-borne pathogens in host-seeking ticks in Anatolia; Initial evidence of putative vectors and pathogens, and footsteps of a secretly rising vector tick, Haemaphysalis parva. Ticks Tick Borne Dis. 2020, 11(3):101373.
[9] Kaenkan W, Nooma W, Chelong IA, et al. Reptile-associated *Borrelia spp.* In Amblyomma ticks, Thailand. Ticks Tick Borne Dis. 2020, 11(1):101315.
[10] Fischer T, Myalkhaa M, Krücken J, et al. Molecular detection of tick-borne pathogens in bovine blood and ticks from Khentii, Mongolia. Transbound Emerg Dis. 2020, 67 (2):111-118.
[11] Ghafar A, Cabezas-Cruz A, Galon C, et al. Bovine ticks harbour a diverse array of microorganisms in Pakistan. Parasit Vectors. 2020;13(1):1.
[12] Rar V, Livanova N, Sabitova Y, et al. *Ixodes persulcatus/pavlovskyi* natural hybrids in Siberia: Occurrence in sympatric areas and infection by a wide range of tick-transmitted agents. Ticks Tick Borne Dis. 2019, 10(6):101254.
[13] Pukhovskaya NM, Morozova OV, Vysochina NP, et al. Prevalence of *Borrelia burgdorferi* sensu lato and *Borrelia miyamotoi* in ixodid ticks in the Far East of Russia. Int J Parasitol Parasites Wildl. 2019, 8:192-202.
[14] Kim CM, Seo JW, Kim DM, et al. Detection of *Borrelia miyamotoi* in *Ixodes nipponensis* in Korea. PLoS One. 2019, 14(7): e0220465.
[15] Duan CJ, Guo Y, Hou XX, et al. Investigation of vector and host infection of Lyme disease in Jinghong, Yunnan. Dis Surv. 2019, 34 (3):246-250.
[16] Jiang BG, Jia N, Jiang JF, et al. *Borrelia miyamotoi* Infections in Humans and

Ticks, Northeastern China. Emerg Infect Dis. 2018, 24(2):236-241.
[17] Hagen RM, Frickmann H, Ehlers J, et al. Presence of *Borrelia spp.* DNA in ticks, but absence of *Borrelia spp.* and of *Leptospira spp.* DNA in blood of fever patients in Madagascar. Acta Trop. 2018, 177:127-134.
[18] Narankhajid M, Yeruult C, Gurbadam A, et al. Some aspects on tick species in Mongolia and their potential role in the transmission of equine piroplasms, *Anaplasma phagocytophilum* and *Borrelia burgdorferi L.* Parasitol Res. 2018, 117(11):3557-3566.
[19] Zheng WQ, Xuan XN, Fu RL, et al. Tick-Borne Pathogens in Ixodid Ticks from Poyang Lake Region, Southeastern China. Korean J Parasitol. 2018, 56(6):589-596.
[20] Khoo JJ, Ishak SN, Lim FS, et al. Detection of a *Borrelia sp.* from *Ixodes granulatus* ticks collected from rodents in Malaysia. J Med Entomol. 2018, 55(6): 1642-1647.
[21] Ye NN, Pei XC, Sun SH. Investigation of the ticks in border trade regions of Heihe. Chin J Zoon. 2018, 34(8):761-767.
[22] Qin LX, Z Q. Investigation on the Infection of *Borrelia Burgdorferi* Carried *Dermacentor sp.* in Yichangerkuang and Xinghuo Pasture in Hailun City of Heilongjiang Province. Nutr Dis. 2018, (8):53-57.
[23] Yang Y, Gao YF, Cao Y, et al. Investigation on tick-borne pathogens in Inner Mongolia Manchuria port area during 2012-2014. Chin J Vector Biol & Control. 2018, 29(2):147-150, 156.
[24] Tang F, Zhou L, Jiang LF, et al. Investigation on *Borrelia burgdorferi* in ticks collected in Qiqian region of Inner Mongolia. Infect Dis Info. 2018, 31(1):31-33.
[25] Liu XY, Gong XY, Zheng C, et al. Molecular epidemiological survey of bacterial and parasitic pathogens in hard ticks from eastern China. Acta Trop. 2017, 167:26-30.
[26] Karasartova D, Gureser AS, Gokce T, et al. Bacterial and protozoal pathogens found in ticks collected from humans in Corum province of Turkey. PLoS Negl Trop Dis. 2018, 12(4): e0006395.
[27] Kuo CC, Lin YF, Yao CT, et al. Tick-borne pathogens in ticks collected from birds in Taiwan. Parasit Vectors. 2017, 10(1):587.
[28] Pan YP, Yang JF Niu QL, et al. Study on *Borrelia burgdorferi sensu lato* and spotted fever group *Rickettsia* in *Ixodes persulcatus* in Heilongjiang Province. Chin Vet Sci. 2017, 47(1):31-37.
[29] Li HY, Zhao SS, Zhang L, et al. Molecular investigation on the genera of *Anaplasma* and *Borrelia* in north region of Xinjiang. J Shihezi Univ：Nat Sci. 2017, 35 (1):108-112.
[30] Sakakibara K, Şen E, Sato K, et al. Detection and Characterization of the Emerging Relapsing Fever Pathogen, *Borrelia miyamotoi*, from the *Ixodes ricinus* Tick in the Rural Trakya (Thrace) Region of Northwestern Turkey. Vector Borne Zoonotic Dis. 2016, 16(12):797-799.
[31] Khasnatinov MA, Danchinova GA, Takano A, et al. Prevalence of *Borrelia miyamotoi* in *Ixodes persulcatus* in Irkutsk City and its neighboring territories, Russia. Ticks Tick Borne Dis. 2016, 7(2):394-397.
[32] Yu PF, Niu QL, Liu ZJ, et al. Molecular epidemiological surveillance to assess emergence and re-emergence of tick-borne infections in tick samples from China

evaluated by nested PCRs. Acta Trop. 2016, 158:181-188.
[33] Yang Y, Wang J, Wang JC, et al. Investigation on tick-borne pathogens in Ganqimaodu port areas on the borders between China and Mongolia, 2012-2013. Chin Front Health Quar. 2016, 39(5):330-332, 347.
[34] Zang F, Wang W, Li L. *Borrelia Burgdorferi* Infection and Genotypes in Ticks and Rodents Collected in Qinghai Province. J Prev Med Chin PLA. 2016, 34(6):799-802.
[35] Han H, Yang Y, Tan KW, et al. Analysis on risk of tick-borne pathogens at Zhangmu port in Tibet. Chin J Vector Biol & Control. 2016, 27 (6):539-541.
[36] Yang J, Wang YM, Ju WD, et al. Investigation on emerging tick-borne pathogens and co-infection in ticks in lava area of Xunke county, Heilongjiang province. Chin J Vector Biol & Control. 2016, 27 (4):341-344.
[37] Hong XK, Liu Y, Xu BL, et al. A study on ticks carrying pathogens at the border of China and Korea. Port Health Control. 2016, 21 (4):57-59.
[38] Liu L, Chen Q, Yang Y, et al. Investigations on Rickettsia in Ticks at the Sino-Russian and Sino-Mongolian Borders, China. Vector Borne Zoonotic Dis. 2015, 15(12): 785-789.
[39] Cheng C, Ju WD, Fu WN, et al. Investigation on compound infection of three tick-borne infectious diseases in vector ticks in Mishan Port, Heilongjiang Province. Chin. J PHM. 2015, 31(3):361-363.
[40] Han H, Shi H, Wang Y, et al. Infection status of *Borrelia burgdorferi sensu lato* and *Anaplasma phagocytophilum* in ticks from the region of Jingpo Lake Mudanjiang. Chin J Hyg Insect ＆ Equip. 2015, 21(2):154-157.
[41] Wang XR, Liu ZJ, Ma RL, et al. Epidemiological Investigation of Animal Lyme Disease in Six Counties of Qinghai Province. Chin J Vet Med. 2015, 51(1):55-56.
[42] Chen Z, Liu Q, Liu JQ, et al. Tick-borne pathogens and associated co-infections in ticks collected from domestic animals in central China. *Parasit Vectors*. 2014, 7:237.
[43] Takano A, Toyomane K, Konnai S, et al. Tick surveillance for relapsing fever spirochete *Borrelia miyamotoi* in Hokkaido, Japan. PLoS One. 2014, 9(8): e104532.
[44] Lee K, Takano A, Taylor K, et al. A relapsing fever group *Borrelia sp.* similar to *Borrelia lonestari* found among wild sika deer (Cervus nippon yesoensis) and *Haemaphysalis spp*. ticks in Hokkaido, Japan. Ticks Tick Borne Dis. 2014, 5(6):841-847.
[45] Hou J, Ling F, Chai C, et al. Prevalence of *Borrelia burgdorferi* sensu lato in ticks from eastern China. Am J Trop Med Hyg. 2014, 92(2):262-266.
[46] Masuzawa T, Masuda S, Fukui T, et al. PCR detection of *Anaplasma phagocytophilum* and *Borrelia burgdorferi* in *Ixodes persulcatus* ticks in Mongolia. Jpn J Infect Dis. 2014, 67(1):47-49.
[46] Orkun Ö, Karaer Z, Çakmak A, et al. Identification of tick-borne pathogens in ticks feeding on humans in Turkey. PLoS Negl Trop Dis. 2014, 8(8): e3067.
[48] Wang W, Li L, Jing LY, et al. Investigation on ticks and the pathogens they could carry in Tianjin. Chin J Hyg Insect ＆ Equip. 2013, 19 (3):220-222.
[49] Sun X, Zhang GL, Liu XM, et al. Investigation of tick species and tick-borne pathogens in Hoxud county of Xinjiang Uyghur Autonomous Region, China. Chin J

Vector Biol & Control. 2013, 24 (1):5-10.
[50] Wang XL, Yang XJ, Guo JH, et al. Distribution and clinical diagnosis of lyme disease in Changchun. Chin J Health Lab Technol. 2013, 23 (9):2160-2161.
[51] Murase Y, Konnai S, Githaka N, et al. Prevalence of Lyme *Borrelia* in *Ixodes persulcatus* ticks from an area with a confirmed case of Lyme disease. J Vet Med Sci. 2013, 75(2):215-218.
[52] Chao LL, Liu LL, Shih CM. Prevalence and molecular identification of *Borrelia* spirochetes in *Ixodes granulatus* ticks collected from Rattus losea on Kinmen Island of Taiwan. Parasit Vectors. 2012, 5:167.
[53] Scholz HC, Margos G, Derschum H, et al. High prevalence of genetically diverse *Borrelia bavariensis*-like strains in *Ixodes persulcatus* from Selenge Aimag, Mongolia. Ticks Tick Borne Dis. 2013, 4(1-2):89-92.
[54] Li YP, Zhou XP. Molecular epidemiological study on natural infection caused by 3 ticks in northerwastern China. Int J Lab Med. 2012, 33 (16):1966-1967.
[55] Ahantarig A, Malaisri P, Hirunkanokpun S, et al. Detection of Rickettsia and a novel *Haemaphysalis shimoga* symbiont bacterium in ticks in Thailand. Curr Microbiol. 2011, 62(5):1496-1502.
[56] Sen E, Uchishima Y, Okamoto Y, et al. Molecular detection of *Anaplasma phagocytophilum* and *Borrelia burgdorferi* in *Ixodes ricinus* ticks from Istanbul metropolitan area and rural Trakya (Thrace) region of north-western Turkey. Ticks Tick Borne Dis. 2011, 2(2):94-98.
[57] Yang Y, Zhang XL, Qiao S, et al. Investigation on tick-borne pathogens at Ganqimaodu port between Sino-Mongolia border. Chin Front Health Quar. 2011, 34(5):339-342.
[58] Huang CM, Wang HC, Lin YC, et al. The presence of *Borrelia valaisiana*-related genospecies in ticks and a rodent in Taiwan. J Microbiol. 2010, 48(6):877-880.
[59] Chao LL, Wu WJ, Shih CM. Molecular detection of *Borrelia valaisiana*-related spirochetes from *Ixodes granulatus* ticks in Taiwan. Exp Appl Acarol. 2010, 52(4):393-407.
[60] Geng Z, Hou XX, Wan KL, et al. Isolation and identification of *Borrelia burgdorferi sensu lato* from ticks in six provinces in China. Chin J Epidemiol. 2010, 31(12):1346-1348.
[61] Niu QL, Guan GQ, Yang JF, et al. Investigations of *Borrelia burgdorferi* infection in ticks by PCR. Chin J Prev Vet Med. 2010, 32(12):984-987.
[62] Chao LL, Wu WJ, Shih CM. First detection and molecular identification of *Borrelia burgdorferi*-like spirochetes in *Ixodes granulatus* ticks collected on Kinmen Island of Taiwan. Am J Trop Med Hyg. 2009, 80(3):389-394.
[63] Wang CS, Wan KL, Guo JH, et al. Investigation of *Borrelia burgdorferi* in media-tick and animals by DFA. Chin Health Lab Technol. 2008, 18 (3):495-496.
[64] Chu CY, Jiang BG, Liu W, et al. Presence of pathogenic *Borrelia burgdorferi sensu lato* in ticks and rodents in Zhejiang, south-east China. J Med Microbiol. 2008, 57(8):980-985.
[65] He H, Hao Q, Hu MX, et al. Investigation on the vectors of *Borrelia burgdorferi* and on the identification of the isolates along China-Russia borter in Eastern

Heilongjiang province, China. Chin J Epidemiol. 2007, 28(1):70-73.
[66] Gao LY, Wang CS, Yang XJ, et al. Studies on the ticks and its transmission Lyme disease in Jilin province. Chin J Hyg Insect ＆ Equip. 2007, 13(1):41-43.
[67] Chu CY, He J, Wang JB, et al. Investigation on *Borrelia burgdorferi sensu lato* in ticks and rodents collected in Da Xing-An Mountains Forest areas of China. Chin J Epidemiol. 2006, 27(8):681-684
[68] Huang HN, Ding Z, He J, et al. Investigation on *Borrelia burgdorferi* infection in ticks and animal from a forest area of Jilin province. Chin J Zoon. 2006, 22(8):785-788.
[69] Hiraoka H, Shimada Y, Sakata Y, et al. Detection of *Borrelia garinii*, *Borrelia tanukii* and *Borrelia* sp. closely related to *Borrelia valaisiana* in *Ixodes* ticks removed from dogs and cats in Japan. Vet Parasitol. 2005, 144(1-2):188-192.
[70] Zhao QM, Wu XM, Zhang PH, et al. Study on the coinfection of three tick-borne infectious diseases in China using polymerase chain reaction method. Chin J Epidemiol. 2005, 26(1):9-13.
[71] Güner ES, Hashimoto N, Takada N, et al. First isolation and characterization of *Borrelia burgdorferi sensu lato* strains from *Ixodes ricinus* ticks in Turkey. J Med Microbiol. 2003, 52(Pt 9):807-813.
[72] Cao WC, Zhao QM, Zhang PH, et al. Prevalence of *Anaplasma phagocytophila* and *Borrelia burgdorferi* in *Ixodes persulcatus* ticks from northeastern China. Am J Trop Med Hyg. 2003, 68(5):547-550.
[73] Wang ZX, Wang DM, Weng CR, et al. Investigation of Lyme Disease in Guizhou Province. Chin J Zoon. 2003, 19(2):113-114.
[74] Takada N, Masuzawa T, Ishiguro F, et al. Lyme disease *Borrelia* spp. in ticks and rodents from northwestern China. Appl Environ Microbiol. 2001, 67(11):5161-5165.
[75] Ishiguro F, Takada N, Masuzawa T, Fukui T. Prevalence of Lyme disease *Borrelia spp.* in ticks from migratory birds on the Japanese mainland. Appl Environ Microbiol. 2000, 66(3):982-986.
[76] Hua MT, Jin ZQ, Gong ZW, et al. Investigation of common ticks carrying bergian spirochetes in the Tiegektilin pastoral area of the Haba River. Chin J Zoon. 2000, 16(1):110,105.
[77] Tian W, Zhang Z, Moldenhauer S, et al. Detection of *Borrelia burgdorferi* from ticks (Acari) in Hebei Province, China. J Med Entomol. 1998, 35(2):95-98.
[78] Takada N, Ishiguro F, Fujita H, Wang HP, Wang JC, Masuzawa T. Lyme disease spirochetes in ticks from northeastern China. J Parasitol. 1998, 84(3):499-504.
[79] Wan KL, Zhang ZF, Dou GL, et al. *Ixodes persulcatus* Plays a Leading Role in the Transmission of *Borrelia burgdorferi sensu lato* to Human in Northern Region of China. Pract Prev Med. 1998, 5 (6):321-324.
[80] Wan KL, Zhang ZF, Zhang JS, et al. Preliminary Investigation on Lyme Disease in Animals in 20 Provinces, Cities and Autonomous Regions of China. Chin J Vector Biol & Control. 1998, 9 (5):36-370.
[81] Miyamoto K, Sato Y, Okada K, Fukunaga M, Sato F. Competence of a migratory bird, red-bellied thrush (Turdus chrysolaus), as an avian reservoir for the Lyme disease spirochetes in Japan. Acta Trop. 1997, 65(1):43-51.
[82] Sato Y, Miyamoto K, Iwaki A, et al. Prevalence of Lyme disease spirochetes in

Ixodes persulcatus and wild rodents in far eastern Russia. Appl Environ Microbiol. 1996, 62(10):3887-3889.
[83] Nakao M, Uchikawa K, Dewa H. Distribution of *Borrelia* species associated with Lyme disease in the subalpine forests of Nagano prefecture, Japan. Microbiol Immunol. 1996, 40(4):307-311.
[84] Takada N, Ishiguro F, Iida H, Yano Y, Fujita H. Prevalence of Lyme *borrelia* in ticks, especially *Ixodes persulcatus* (Acari: Ixodidae), in central and western Japan. J Med Entomol. 1994, 31(3):474-478.
[85] Mo YM, Wang GQ, Chen XY, et al. Investigation of Lyme Disease in Yingshan County, Hubei Province. Publ Health Chin. 1994, 10(3):104-105.
[86] Nakao M, Miyamoto K, Uchikawa K, Fujita H. Characterization of *Borrelia burgdorferi* isolated from *Ixodes persulcatus* and *Ixodes ovatus* ticks in Japan. Am J Trop Med Hyg. 1992, 47(4):505-511.
[87] Miyamoto K, Nakao M, Uchikawa K, Fujita H. Prevalence of Lyme *borreliosis* spirochetes in ixodid ticks of Japan, with special reference to a new potential vector, *Ixodes ovatus* (Acari: Ixodidae). J Med Entomol. 1992, 29(2):216-220.
[88] Du Y, Tang SY, Wu XM, et al. Investigation on vector of Lyme disease in natural foci of Weihe forest area of Heilongjiang Province. Med animal control. 1992, 8(1):33-35.
[89] Zhang QE, Zhang PH, Li SQ, Li RY, Pei DK. Investigation of Lyme disease in Xinjiang. Chin Med J (Engl). 1991, 104(3):244-246.
[90] Ai CX, Hu RJ, Hyland KE, et al. Epidemiological and aetiological evidence for transmission of Lyme disease by adult *Ixodes persulcatus* in an endemic area in China. Int J Epidemiol. 1990, 19(4):1061-1065.
[91] Li SQ, Wang FZ, Zhang QE, et al. Investigation on natural foci of Lyme disease in western Tianshan forest area. Post-land health. 1990, 11 (1):47-48.

.

Supplementary Material 4: Study characteristic

Study	Authors	Event	No	Country	Method	Phase	Capture month	Genus	No.infection/No.examined
1	Gao Y (2021)	10	774	China	PCR	-	4-9	264 *I. persulcatus,* 127 *D. nuttalli,* 78 *D. silvarum,* 275 *H. longicornis*	7/264 *I. persulcatus,* 1/127 *D. nuttalli,* 1/78 *D. silvarum,* 1/275 *H. longicornis*
2	Seo HJ (2021)	15	380	South Korea	PCR	adult, larva, nymph	-	40 *I. nipponensis,* 230 *I. turdus,* 74 *H. flava,* 2 *H. formosensis,* 29 *H. longicornis,* 4 *H. phasiana,* 1 *A. testudinarium*	0/40 *I. nipponensis,* 14/230 *I. turdus,* 1/74 *H. flava,* 0/2 *H. formosensis,* 0/29 *H. longicornis,* 0/4 *H. phasiana,* 0/1 *A. testudinarium*
3	Wang Q (2021)	18	250	China	PCR	adult, nymph	4-6	120 *D. silvarum,* 21 *D. nuttalli,* 47 *H. concinna,* 38 *H. japonica,* 42 *I. persulcatus*	1/120 *D. silvarum,* 0/21*D. nuttalli,* 2/47 *H. concinna,* 2/38 *H. japonica,* 13/42 *I. persulcatus*
4	Seo MG (2020)	6	329	South Korea	PCR	adult, nymph	5-9	14 *I. nipponensis,* 282 *H. longicornis,* 33 *H. flava*	2/14 *I. nipponensis,* 3/282 *H. longicornis,* 1/33 *H. flava*
5	Kim SY (2020)	248	738	South Korea	PCR	larva, nymph	3-5, 9-11	193 *I. nipponensis,* 96 *I. angustus,* 440 *I. spp.,* 9 *H. longicornis*	109/193 *I. nipponensis,* 3/96 *I. angustus,* 135/440 *I. spp.,* 1/9 *H. longicornis*
6	Nakayama S (2020)	2	561	Japan	PCR	adult, larva, nymph	1-12	3 *I. turdus,* 5 *I. ovatus,* 22 *A. testudinarium,* 213 *H. flava,* 78 *H. formosensis,* 24 *H. kitaokai,* 137 *H. longicornis,* 15 *H. hystricis,* 28 *H. megaspinosa*	1/3 *I. turdus,* 0/5 *I. ovatus,* 0/22 *A. testudinarium,* 0/213 *H. flava,* 0/78 *H. formosensis,* 0/24 *H. kitaokai,* 0/137 *H. longicornis,* 0/15 *H. hystricis,* 1/28 *H. megaspinosa*
7	Seto J (2020)	12	164	Japan	PCR	adult	3-7	9 *I. persulcatus,* 95 *I. ovatus,* 4 *I. tanuki,* 37 *I. nipponensis,* 7 *H. longicornis,* 10 *H. flava,* 2 *H. japonica*	1/9 *I. persulcatus,* 11/95 *I. ovatus,* 0/37 *I. nipponensis,* 0/4 *I. tanuki,* 0/7 *H. longicornis,* 0/10 *H. flava,* 0/2 *H. japonica*
8	Orkun Ö (2020)	3	1019	Turkey	PCR	adult, nymph	-	30 *I. ricinus,* 739 *H. parva,* 16 *H. sulcata,* 3 *H. punctata,* 105 *H. marginatum,* 11 *H. excavatum,* 9 *H. aegyptium,* 41 *D. marginatus,* 2 *D. reticulatus,* 9 *Rh. turanicus*	3/30 *I. ricinus,* 0/739 *H. parva,* 0/16 *H. sulcata,* 0/3 *H. punctata,* 0/105 *H. marginatum,* 0/11 *H. excavatum,* 0/9 *H. aegyptium,* 0/41 *D. marginatus,* 0/2 *D. reticulatus,* 0/9 *Rh. turanicus*

9	Kaenkan W (2020)	20	127	Thailand	PCR	adult, nymph	-	28 *A. helvolum,* 62 *A. varanense,* 31 *A. geoemydae,* 6 *A. spp*	1/28 *A. helvolum,* 17/62 *A. varanense,* 2/31 *A. geoemydae,* 0/6 *A. spp*
10	Fischer T (2020)	1	310	Mongolia	PCR	adult	5-6	310 *D. nuttalli*	1/310 *D. nuttalli*
11	Ghafar A (2020)	15	234	Pakistan	PCR	-	9-11	212 *Hy. Anatolicum,* 3 *Hy. Hussaini,* 1 *Hy. Scupense,* 17 *Rh. Microplus,* 1 *Rh. annulatus*	14/212 *Hy. Anatolicum,* 0/3 *Hy. Hussaini,* 0/1 *Hy. Scupense,* 1/17 *Rh. Microplus,* 0/1 *Rh. annulatus*
12	Rar V (2019)	742	1822	Russia (Siberia)	PCR	-	5-6	668 *I. persulcatus,* 1154 *I. pavlovskyi*	252/668 *I. persulcatus,* 490/1154 *I. pavlovskyi*
13	Pukhovskaya NM (2019)	605	2158	Russia (Siberia)	PCR	-	-	1599 *I. persulcatus,* 481 *H. concinna,* 49 *H. japonica,* 29 *D. silvarum*	582/1599 *I. persulcatus,* 14/481 *H. concinna,* 7/49 *H. japonica,* 2/29 *D. silvarum*
14	Kim CM (2019)	5	484	South Korea	PCR	adult, larva, nymph	4-9	25 *I. nipponensis,* 417 *H. longicornis,* 42 *H. flava*	5/25 *I. nipponensis,* 0/417 *H. longicornis,* 0/42 *H. flava*
15	Duan CJ (2019)	3	724	China	other	-	3	724 *R(B). microplus*	3/724 *R(B). microplus*
16	Jiang BG (2018)	20	692	China	PCR	adult	-	627 *I. persulcatus,* 36 *H. concinna,* 29 *D. silvarum*	19/627 *I. persulcatus,* 1/36 *H. concinna,* 0/29 *D. silvarum*
17	Hagen RM (2018)	23	403	South Korea	PCR	adult, nymph	-	255 *A. variegatum,* 148 *R. microplus*	21/255 *A. variegatum,* 2/148 *R. microplus*
18	Narankhajid M (2018)	9	360	Mongolia	PCR	adult	-	16 *I. persulcatus,* 402 *D. nuttalli,* 44 *D. marginatus,* 6 *D. silvarum,* 60 *H. asiaticum*	2/7 *I. persulcatus,* 6/272 *D. nuttalli,* 0/35 *D. marginatus, 0/2 D. silvarum,* 1/44 *H. asiaticum*
19	Zheng WQ (2018)	3	311	China	PCR	adult, larva, nymph	-	16 *I. granulatus,* 1 *I. acuminatus,* 4 *I. sinensis,* 173 *H. longicornis,* 21 *H. flava,* 77 *R. microplus,* 5 *H. phasiana,* 9 *R. sanguineus,* 3 *R. haemaphysaloides,* 1 *A. testudinarium,* 1*D. auratus*	3/16 *I. granulatus,* 0/1 *I. acuminatus, 0/4 I. sinensis,* 0/173 *H. longicornis, 0/21 H. flava, 0/77 R. microplus,* 0/5 *H. phasiana*, 0/9 *R. sanguineus, 0/3 R. haemaphysaloides, 0/1 A. testudinarium, 0/1 D. auratus*
20	Khoo JJ (2018)	72	156	Malaysia	PCR	-	1-12	122 *I. granulatus,* 24 *D. spp,* 3 *A. spp*	72/122 *I. granulatus,* 0/24 *D. spp,* 0/3 *A. spp*
21	Ye NN (2018)	0	1111	China	PCR	-	4-10	1054 *D. silvarum,* 236 *H. concinna,* 53 *I. persulcatus*	0/1054 *D. silvarum,* 0/236 *H. concinna,* 0/53 *I. persulcatus*

22	Qin LX (2018)	51	96	China	PCR	-	-	96 *D. spp*	51/96 *D. spp*
23	Yang Y (2018)	20	388	China	PCR	-	5-7	267 *I. persulcatus,* 559 *D. nuttalli*	0/125 *I. persulcatus*，20/263 *D. nuttalli*
24	Tang F (2018)	86	320	China	PCR	-	4-6	293 *I. persulcatus,* 22 *D. silvarum,* 5 *H. concinna*	85/293 *I. persulcatus,* 1/22 *D. silvarum,* 0/5 *H. concinna*
25	Liu XY (2017)	0	500	China	PCR	adult	1-12	500 *H. longicornis*	0/500 *H. longicornis*
26	Karasartova D (2017)	1	322	Turkey	PCR	-	-	4 *I. ricinus,* 164 *H. marginatum,* 5 *H. excavatum,* 1 *H. aegyptium,* 41 *H. Parva,* 6 *H. Punctata,* 1 *H. Sulcata,* 46 *H. spp.,* 34 *R. turanicus,* 3 *R. bursa,* 17 *D. marginatus*	1/4 *I. ricinus,* 0/164 *H. marginatum,* 0/5 *H. excavatum,* 0/1 *H. aegyptium,* 0/41 *H. Parva,* 0/6 *H. Punctata,* 0/1 *H. Sulcata,* 0/46 *H. spp.,* 0/34 *R. turanicus,* 0/3 *R. bursa,* 0/17 *D. marginatus*
27	Kuo CC (2017)	5	85	China	PCR	adult, larva, nymph	1-12	42 *H. doenitzi,* 1 *H. flava,* 2 *H. formosensis,* 12 *H. hystricis,* 21 *H. ornithophila,* 17 *H. wellingtoni,* 17 *I. columnae,* 6 *A. spp* 5 *I. granulatus,* 1 *I. nipponensis,* 4 *I. turdus,* 1 *R. haemaphysaloides,* 4 *I. spp,* 6*H. spp,*	3/4 *I. granulatus,* 2/5 *I. turdus, 0/*24 *H. doenitzi, 0/*1 *H. flava, 0/*1 *H. formosensis, 0/*9 *H. hystricis, 0/*6 *H. ornithophila, 0/*8 *H. wellingtoni, 0/*17 *I. columnae, 0/*1 *I. nipponensis, 0/*1 *R. haemaphysaloides, 0/*3 *H. spp.,* 0/5 *A. spp.*
28	Pan YP (2017)	5	25	China	PCR	adult	4-5	25 *I. persulcatus*	5/25 *I. persulcatus*
29	Li HY (2017)	97	546	China	PCR	-	4-5	210 *H. asiaticum*, 209 *H. punctate,* 61 *D. nuttalli,* 40 *D. marginatus,* 26 *R. turanicus*	-
30	Sakakibara K (2016)	1	248	Turkey	PCR	adult	6	248 *I. ricinus*	1/248 *I. ricinus*
31	Khasnatinov MA (2016)	90	445	Russia (Siberia)	PCR	adult	-	445 *I. persulcatus*	0/445 *I. persulcatus*
32	Yu PF (2016)	156	849	China	PCR	-	-	34 *H. bispinosa,* 165 *D. silvarum,*	11/ 34 *H. bispinosa,* 3/165 *D. silvarum,*

								213 *R. (B.) microplus,* 125 *H. qinghaiensis,* 186 *R. sanguineus* ,84 *H. longicornis,*<br>42 *I. persulcatus*	18/213 *R. (B.) microplus,* 18/42 *I. persulcatus*<br>3/125 *H. qinghaiensis,* 65/84 *H. longicornis*<br>38/186 *R. sanguineus*
33	Yang Y (2016)	0	178	China	PCR	-	-	178 *H. asiaticum*	0/178 *H. asiaticum*
34	Zang F (2016)	97	618	China	PCR	adult	3-4	249 *D. nuttalli*, 152 *H. garhwalensis,* 217 *H. qinghaiensis*	28/249 *D. nuttalli,* 22/152 *H. garhwalensis,* 47/217 *H. qinghaiensis*
35	Han H (2016)	3	232	China	PCR	-	-	69 *H. nepalensis,* 5 *H. formosensis,* 86 *R. microplus,* 72 *unidentified*	3/86 *R. microplus,* 0/69 *H. nepalensis,* 0/5 *H. formosensis,* 0/72 unidentified
36	Yang J (2016)	0	257	China	PCR	-	4-6	106 *H. concinna,* 84 *I. persulcatus,* 66 *D. silvarum,* 1 *H. japonica*	0/106 *H. concinna,* 0/84 *I. persulcatus,* 0/66 *D. silvarum,* 0/1 *H. japonica*
37	Hong XK (2016)	2	90	China	PCR	-	3-10	-	-
38	Liu L (2015)	0	292	China	PCR	adult	5-6	113 *I. persulcatus,* 179 *D. nuttalli*	0/113 *I. persulcatus,* 0/179 *D. nuttalli*
39	Cheng C (2015)	4	44	China	PCR	-	4-6	8 *I. persulcatus,* 34 *H. concinna,* 2 *D. silvarum*	3/8 *I. persulcatus,* 1/34 *H. concinna*, 0/2 *D. silvarum*
40	Han H (2015)	12	643	China	PCR	-	5	573 *H. concinna,* 70 *I. persulcatus*	1/573 *H. concinna,* 11/70 *I. persulcatus*
41	Wang XR(2015)	53	227	China	other	-	3-8	117 *H. qinghaiensis,* 110 *D. nuttalli*	28/117 *H. qinghaiensis,* 25/110 *D. nuttalli*
42	Chen Z (2014)	0	308	China	PCR	adult, larva, nymph	4-12	298 *H. longicornis,* 10 *R. microplus*	0/298 *H. longicornis,* 0/10 *R. microplus*
43	Takano A (2014)	77	4325	Japan	PCR	adult	4-7	3532 *I. persulcatus,* 117 *I. pavlovskyi,* 676 *I. ovatus*	71/3532 *I. persulcatus,* 5/117 *I. pavlovskyi,*<br>1/676 *I. ovatus*
44	Lee K (2014)	6	670	Japan	PCR	adult, nymph	5-9	670 *H. spp.*	6/670 *H. spp.*
45	Hou J (2014)	21	447	China	PCR	adult	1-12	212 *H. longicornis,* 20 *R. haemaphysaloides,* 29 *A. testudinarium,* 94 *I. sinensis,*<br>20 *B. microplus,* 19 *H. yeni,*<br>9 *D. taiwanensis,* 5 *H. hystricis,*	11/212 *H. longicornis,* 0/20 *R. haemaphysaloides,* 1/29 *A. testudinarium,* 1/94 *I. sinensis,*<br>1/20 *B. microplus,* 0/19 *H. yeni,*<br>0/9 *D. taiwanensis,* 0/5 *H. hystricis,*

								1 *H. asiaticum,*36 *I. granulates,* 2 *I. ovatus*	0/1 *H. asiaticum,* 7/36 *I. granulates,* 0/2 *I. ovatus*
46	Masuzawa T (2014)	71	129	Mongolia	PCR	-	6	129 *I. persulcatus*	71/129 *I. persulcatus*
47	Orkun O (2014)	6	169	Turkey	PCR	-	1-12	17 *H. excavatum,* 30 *H. marginatum,* 6 *Hy. spp,* 35 *H. Parva,* 28 *R. turanicus,* 25 *D. marginatus,* 16 *H. aegyptium,* 3 *H. punctata,* 3 *R. bursa,* 3 *R. sanguineus,* 2 *I. ricinus,* 1 *H. spp.*	1/17 *H. excavatum,* 1/30 *H. marginatum,* 2/6 *Hy. spp,* 2/35 *H. Parva,* 0/28 *R. turanicus,* 0/25 *D. marginatus,* 0/16 *H. aegyptium,* 0/3 *H. punctata,* 0/3 *R. bursa,* 0/3 *R. sanguineus,* 0/2 *I. ricinus,* 0/1 *H. spp.*
48	Wang W (2013)	0	197	China	other	-	-	58 *I. persulcatus,* 139 *H. longicornis*	0/58 *I. persulcatus,* 0/139 *H. longicornis*
49	Sun X (2013)	18	367	China	PCR	-	4	174 *H. asiaticum,* 138*R. pumilio,* 14 *R. turanicus,* 41 *D. marginatus*	3/174 *H. asiaticum,* 15/138 *R. pumilio,* 0/14 *R. sturanicus,* 0/41 *D. marginatus*
50	Wang XL (2013)	229	542	China	PCR	-	-	-	-
51	Murase Y (2012)	119	338	Japan	PCR	-	4	284 *I. persulcatus,* 41 *I. ovatus,* 13 *H. longicornis*	98/284 *I. persulcatus,* 8/41 *I. ovatus,* 1/13 *H. longicornis*
52	Chao LL (2012)	123	261	China	PCR	adult, larva	-	261 *I. granulatus*	123/ 261 *I. granulatus*
53	Scholz HC (2012)	91	372	Mongolia	PCR	adult	5	372 *I. persulcatus*	91/372 *I. persulcatus*
54	Li YP (2012)	304	2460	China	PCR	-	4-6	-	-
55	Ahantarig A (2011)	0	166	Thailand	PCR	adult, larva, nymph	-	166 *H. spp.*	0/166 *H. spp.*
56	Sen E (2011)	10	24	Turkey	PCR	-	6	24 *I. ricinus*	10/24 *I. ricinus*
57	Yang Y (2011)	5	162	China	PCR	-	5	162 *H. asiaticum*	5/162 *H. asiaticum*
58	Huang CM (2010)	23	113	China	PCR	adult, larva, nymph	-	113 *I. granulatus*	23/113 *I. granulatus*
59	Chao LL (2010)	27	147	China	PCR	adult	-	147 *I. granulatus*	27/147 *I. granulatus*

60	Geng Z (2010)	233	1515	China	other	-	-	-	-
61	Niu QL (2010)	94	631	China	PCR	-	4-7	264 *D. spp.,* 173 *H. spp.,* 185 *R. Curtice,* 45 *R. spp.*	10/264 *D.spp,* 11/173 *H. spp.,* 59/185 *R. Curtice,* 14/45 *R. spp.*
62	Chao LL (2009)	20	78	China	PCR	adult	-	78 *I. granulatus*	20/78 *I. granulatus*
63	Wang CS(2009)	551	1405	China	other	-	-	1239 *I. persulcatus,* 110 *H. concinna*	444/1239 *I. persulcatus,* 9/110 *H. concinna*
64	Chu CY (2008)	41	182	China	PCR	adult	10-12	4 *I. vespertlionis,* 1 *I. hyatti,* 2 *H. concinna,* 114 *H. longicornis,* 40 *H. warburconi,* 21 *R. microplus*	0/4 *I. vespertlionis,* 0/1 *I. hyatti,* 1/2 *H. concinna,* 32/114 *H. longicornis,* 5/40 *H. warburconi,* 3/21 *R. microplus*
65	He H (2007)	39	105	China	other	-	-	2065 *I. persulcatus,* 2438 *D. silvarum,* 1848 *H. concinna,* 203 *H. japonica*	-
66	Gao LY (2007)	631	1799	China	other	-	-	1799 *I. persulcatus,*	631/1799 *I. persulcatus*
67	Chu CY (2006)	299	1624	China	PCR	-	5-7	1336 *I. persulcatus,* 144 *D. silvarum,* 144 *H. concinna*	293/1336 *I. persulcatus,* 6/144 *D. silvarum,* 0/144 *H. concinna*
68	Huang HM (2006)	137	427	China	PCR	-	4-5	100 *I.persulcatus,* 327 *D.silvarum*	36/100 *I.persulcatus,* 101/327 *D.silvarum*
69	Hiroko H (2005)	6	1269	Japan	PCR	-	-	-	-
70	Zhao QM (2005)	39	2035	China	PCR	-	4-10	-	-
71	Güner ES(2003)	12	299	Turkey	other	-	5	299 *I. ricinus*	12/299 *I. ricinus*
72	Cao WC (2003)	454	1345	China	PCR	-	6-8	1345 *I. persulcatus*	454/1345 *I. persulcatus*
73	Wang ZX (2003)	42	274	China	other	-	1-12	-	-
74	Takada N (2001)	22	137	China	PCR	adult, larva, nymph	5	66 *I. persulcatus*	26/66 *I. persulcatus*
75	Ishiguro F (2000)	28	303	Japan	PCR	adult, larva, nymph	1-5, 9-12	343 *H. flava,* 6 *H. longicornis,* 1 *I. columnae,* 9 *I. persulcatus,*	0/2 *H. flava,* 0/2 *H. longicornis,* 0/1 *H. phasiana,* 0/11 *I. turdus*

								1 *H. phasiana,* 15 *I. turdus,* 2 *unidentified*	
76	Hua MT (2000)	10	62	China	other	-	5	1543 *D. silvarum,* 1099 *D. niveus,* 383 *D. nuttalli,* 58 *D. marginatus,* 3 *H. concinna,* 2 *H. campanulata,* 1 *H. detritum,* 1 *I. persulcatus*	6/32 *D. silvarum,* 4/30 *D. niveus*
77	Tian W (1998)	9	1118	China	PCR	adult, larva, nymph	5-9	947 *H. japonicum* and *H. longicornis,* 158 *I. persulcatus,* 13 *D. silvarum*	4/947 *H. japonicum* and *H. longicornis,* 5/158 *I. persulcatus,* 0/13 *D. silvarum*
78	Takada N (1998)	59	128	China	PCR	adult	5	21 *I. nipponensis,* 287 *I. persulcatus,* 1 *I. pavlovskyi,* 42 *H. concinna,* 7 *H. flava,* 15 *H. douglasi,* 45 *H. longicornis,* 6 *H. megaspinosa,* 2 *H. punctata,* 59 *H. spp,* 12 *D. silvarum,* 7 *D. silvarum*	-
79	Wan KL (1998)	166	623	China	other	-	-	-	-
80	Wan KL (1998)	128	820	China	other	-	-	-	-
81	Miyamoto K (1997)	7	47	Japan	PCR	adult, larva, nymph	10	113 *I. persulcatus*	7/47 *I. persulcatus*
82	Sato Y (1996)	225	846	Russia (Siberia)	other	adult	5-6	846 *I. persulcatus*	225/846 *I. persulcatus*
83	NAKAO M (1996)	114	300	Japan	PCR	adult	5-7	300 *l. persulcatus*	112/300 *l. persulcatus*
84	Takada N (1994)	81	528	Japan	other	adult, larva	4-7	1 *A. testudinarium,* 8 *D. taiwanensis,* 40 *H. flava,* 6 *H. japonica,* 23*H. kitaokai,* 7 *H. longicornis,* 12 *H. megaspinosa,* 2 *I. angustus,* 58 *I. monospinosus,* 6 *I. nipponensis,* 193 *I. ovatus,* 85 *I. persulcatus,* 2 *I. spp.*	37/193 *I. ovatus,* 11/85 *I. persulcatus,* 0/1 *A. testudinarium,* 0/8 *D. taiwanensis,* 1/40 *H. flava,* 0/6 *H. japonica,* 0/23 *H. kitaokai,* 0/12 *H. megaspinosa,* 0/58 *I. monospinosus*
85	Mo YM (1994)	5	100	China	other	-	-	308 *R(B). microplus,* 136 *H. hystricis,* 40 *H. bispinosa*	0/40 *R(B). microplus,* 2/40 *H. hystricis,* 3/20 *H, bispinosa*
86	NAKAO M (1992)	225	1464	Japan	other	adult	1-12	777 *I. persukatus,* 687 *I. ovatus*	129/777 *I. persukatus,* 162/687 *I. ovatus*
87	Miyamoto K (1992)	117	909	Japan	other	adult, larva	4-8	273 *I. ovatus,* 792 *I. persulcatus*	41/273 *I. ovatus,* 130/792 *I. persulcatus*

88	Du Y (1992)	215	846	China	other	-	4-7	766 *I. persulcatus,* 62 *H. japonica,* 18 *D. silvarum*	215/766 *I. persulcatus,* 0/62 *H. japonica,* 18 *D. silvarum*
89	Zhag QN (1991)	42	150	China	other	adult	4-7	150 *I. persulcatus,* 105 *D. marginatus,* 45 *H. spp.*	42/150 *I. persulcatus,* 0/105 *D. marginatus,* 0/45 *H. spp*
90	Ai CX (1990)	42	582	China	other	adult	4-8	567 *l. persulcatus,* 15 *H. concinna*	-
91	Li SQ (1990)	39	343	China	PCR	adult, larva, nymph	4-7	-	-

Supplementary Material 5: Asia prevalence of B. burgdorferi in Ixodidae by subgroup

Study	Events	Total	Proportion	95%−CI	Weight
**country = China**					
Gao Y(2021)	10	774	0.013	[0.006; 0.024]	1.1%
Wang Q(2021)	18	250	0.072	[0.043; 0.111]	1.1%
Duan CJ (2019)	3	724	0.004	[0.001; 0.012]	1.1%
Jiang BG(2018)	20	692	0.029	[0.018; 0.044]	1.1%
Zheng WQ (2018)	3	311	0.010	[0.002; 0.028]	1.1%
Ye NN (2018)	0	1111	0.000	[0.000; 0.003]	1.1%
Qin LX (2018)	51	96	0.531	[0.427; 0.634]	1.1%
Yang Y (2018)	20	388	0.052	[0.032; 0.078]	1.1%
Tang F (2018)	86	320	0.269	[0.221; 0.321]	1.1%
Liu XY(2017)	0	500	0.000	[0.000; 0.007]	1.1%
Kuo CC (2017)	5	85	0.059	[0.019; 0.132]	1.1%
Pan YP (2017)	5	25	0.200	[0.068; 0.407]	0.9%
Li HY (2017)	97	546	0.178	[0.146; 0.212]	1.1%
Yu PF (2016)	156	849	0.184	[0.158; 0.211]	1.1%
Yang Y (2016)	0	178	0.000	[0.000; 0.021]	1.1%
Zhang F (2016)	97	618	0.157	[0.129; 0.188]	1.1%
Han H (2016)	3	232	0.013	[0.003; 0.037]	1.1%
Yang J (2016)	0	257	0.000	[0.000; 0.014]	1.1%
Hong XK (2016)	2	90	0.022	[0.003; 0.078]	1.1%
Liu L(2015)	0	292	0.000	[0.000; 0.013]	1.1%
Chen C (2015)	4	44	0.091	[0.025; 0.217]	1.0%
Han H (2015)	12	643	0.019	[0.010; 0.032]	1.1%
Wang XR (2015)	53	227	0.233	[0.180; 0.294]	1.1%
Chen Z(2014)	0	308	0.000	[0.000; 0.012]	1.1%
Hou J (2014)	21	447	0.047	[0.029; 0.071]	1.1%
Wang W (2013)	0	197	0.000	[0.000; 0.019]	1.1%
Sun X (2013)	18	367	0.049	[0.029; 0.076]	1.1%
Wang XL (2013)	229	542	0.423	[0.381; 0.465]	1.1%
Chao LL (2012)	123	261	0.471	[0.409; 0.534]	1.1%
Li YP (2012)	304	2460	0.124	[0.111; 0.137]	1.1%
Yang Y (2011)	5	162	0.031	[0.010; 0.071]	1.1%
Huang CM (2010)	23	113	0.204	[0.134; 0.290]	1.1%
Chao LL (2010)	27	147	0.184	[0.125; 0.256]	1.1%
Geng Z (2010)	233	1515	0.154	[0.136; 0.173]	1.1%
Niu QL (2010)	94	631	0.149	[0.122; 0.179]	1.1%
Chao LL (2009)	20	78	0.256	[0.164; 0.368]	1.1%
Wang CS (2009)	551	1405	0.392	[0.367; 0.418]	1.1%
Chu CY(2008)	41	182	0.225	[0.167; 0.293]	1.1%
He H (2007)	39	105	0.371	[0.279; 0.471]	1.1%
Gao LY (2007)	631	1799	0.351	[0.329; 0.373]	1.1%
Chu CY (2006)	299	1624	0.184	[0.166; 0.204]	1.1%
Huang HM (2006)	137	427	0.321	[0.277; 0.367]	1.1%
Zhao QM (2005)	39	2035	0.019	[0.014; 0.026]	1.1%
Cao WC (2003)	454	1345	0.338	[0.312; 0.364]	1.1%
Wang ZX (2003)	42	274	0.153	[0.113; 0.201]	1.1%
Takada N (2001)	22	137	0.161	[0.103; 0.233]	1.1%
Hua MT (2000)	10	62	0.161	[0.080; 0.277]	1.0%
Tian W (1998)	9	1118	0.008	[0.004; 0.015]	1.1%
Takada N (1998)	59	128	0.461	[0.372; 0.551]	1.1%
Wan KL (1998)	166	623	0.266	[0.232; 0.303]	1.1%
Wang KL (1998)	128	820	0.156	[0.132; 0.183]	1.1%
Mo YM (1994)	5	100	0.050	[0.016; 0.113]	1.1%
Du Y (1992)	215	846	0.254	[0.225; 0.285]	1.1%
Zhag QN (1991)	42	150	0.280	[0.210; 0.359]	1.1%
Ai CG (1990)	42	582	0.072	[0.053; 0.096]	1.1%
Li SQ (1990)	39	343	0.114	[0.082; 0.152]	1.1%
**Random effects model**		**30585**	**0.115**	**[0.080; 0.154]**	**61.5%**
Heterogeneity: I^2 = 99%, τ^2 = 0.0483, p = 0					
**country = Japan**					
Nakayama S(2020)	2	561	0.004	[0.000; 0.013]	1.1%
Seto J(2020)	12	164	0.073	[0.038; 0.124]	1.1%
Takano A(2014)	77	4325	0.018	[0.014; 0.022]	1.1%
Lee K(2014)	6	670	0.009	[0.003; 0.019]	1.1%
Murase Y (2012)	119	338	0.352	[0.301; 0.406]	1.1%
Hiroko H (2005)	6	1269	0.005	[0.002; 0.010]	1.1%
Ishiguro F (2000)	28	303	0.092	[0.062; 0.131]	1.1%
Miyamoto K (1997)	7	47	0.149	[0.062; 0.283]	1.0%
NAKAO M (1996)	114	300	0.380	[0.325; 0.438]	1.1%
Takada N (1994)	81	528	0.153	[0.124; 0.187]	1.1%
NAKAO M (1992)	225	1464	0.154	[0.136; 0.173]	1.1%
Miyamoto K (1992)	117	909	0.129	[0.108; 0.152]	1.1%
**Random effects model**		**10878**	**0.096**	**[0.044; 0.167]**	**13.3%**
Heterogeneity: I^2 = 99%, τ^2 = 0.0338, p < 0.01					
**country = Malaysia**					
Khoo JJ (2018)	72	156	0.462	[0.382; 0.543]	1.1%
**Random effects model**		**156**	**0.462**	**[0.384; 0.540]**	**1.1%**
Heterogeneity: not applicable					
**country = Mongolia**					
Fischer T(2020)	1	310	0.003	[0.000; 0.018]	1.1%
Narankhajid M (2018)	9	360	0.025	[0.011; 0.047]	1.1%
Masuzawa T (2014)	71	129	0.550	[0.460; 0.638]	1.1%
Scholz HC (2012)	91	372	0.245	[0.202; 0.292]	1.1%
**Random effects model**		**1171**	**0.145**	**[0.008; 0.407]**	**4.4%**
Heterogeneity: I^2 = 99%, τ^2 = 0.0933, p < 0.01					
**country = Pakistan**					
Ghafar A(2020)	15	234	0.064	[0.036; 0.104]	1.1%
**Random effects model**		**234**	**0.064**	**[0.036; 0.099]**	**1.1%**
Heterogeneity: not applicable					
**country = Russia(Siberia)**					
Rar V(2019)	742	1822	0.407	[0.385; 0.430]	1.1%
Pukhovskaya NM(2019)	605	2158	0.280	[0.261; 0.300]	1.1%
Khasnatinov MA(2016)	90	445	0.202	[0.166; 0.243]	1.1%
Sato Y(1996)	225	846	0.266	[0.236; 0.297]	1.1%
**Random effects model**		**5271**	**0.288**	**[0.210; 0.372]**	**4.5%**
Heterogeneity: I^2 = 97%, τ^2 = 0.0081, p < 0.01					
**country = South Korea**					
Seo HJ(2021)	15	380	0.039	[0.022; 0.064]	1.1%
Seo MG(2020)	6	329	0.018	[0.007; 0.039]	1.1%
Kim SY(2020)	248	738	0.336	[0.302; 0.371]	1.1%
Kim CM(2019)	5	484	0.010	[0.003; 0.024]	1.1%
Hagen RM(2018)	23	403	0.057	[0.037; 0.084]	1.1%
**Random effects model**		**2334**	**0.066**	**[0.002; 0.207]**	**5.6%**
Heterogeneity: I^2 = 99%, τ^2 = 0.0585, p < 0.01					
**country = Thailand**					
Kaenkan W(2020)	20	127	0.157	[0.099; 0.233]	1.1%
Ahantarig A(2011)	0	166	0.000	[0.000; 0.022]	1.1%
**Random effects model**		**293**	**0.041**	**[0.000; 0.322]**	**2.2%**
Heterogeneity: I^2 = 98%, τ^2 = 0.0815, p < 0.01					
**country = Turkey**					
Orkun O(2020)	3	1019	0.003	[0.001; 0.009]	1.1%
Karasartova D(2017)	1	322	0.003	[0.000; 0.017]	1.1%
Sakakibara K(2016)	1	248	0.004	[0.000; 0.022]	1.1%
Orkun O (2014)	6	169	0.036	[0.013; 0.076]	1.1%
Sen E (2011)	10	24	0.417	[0.221; 0.634]	0.9%
Güner ES(2003)	12	299	0.040	[0.021; 0.069]	1.1%
**Random effects model**		**2081**	**0.028**	**[0.007; 0.065]**	**6.5%**
Heterogeneity: I^2 = 92%, τ^2 = 0.0103, p < 0.01					
**Random effects model**		**53003**	**0.111**	**[0.083; 0.142]**	**100.0%**
Heterogeneity: I^2 = 99%, τ^2 = 0.0503, p = 0					
Residual heterogeneity: I^2 = 99%, p = 0					

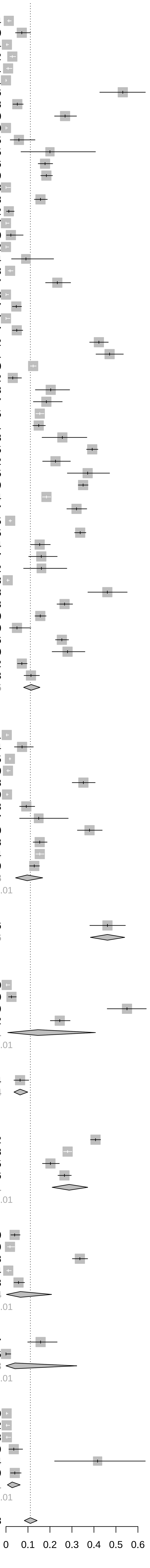

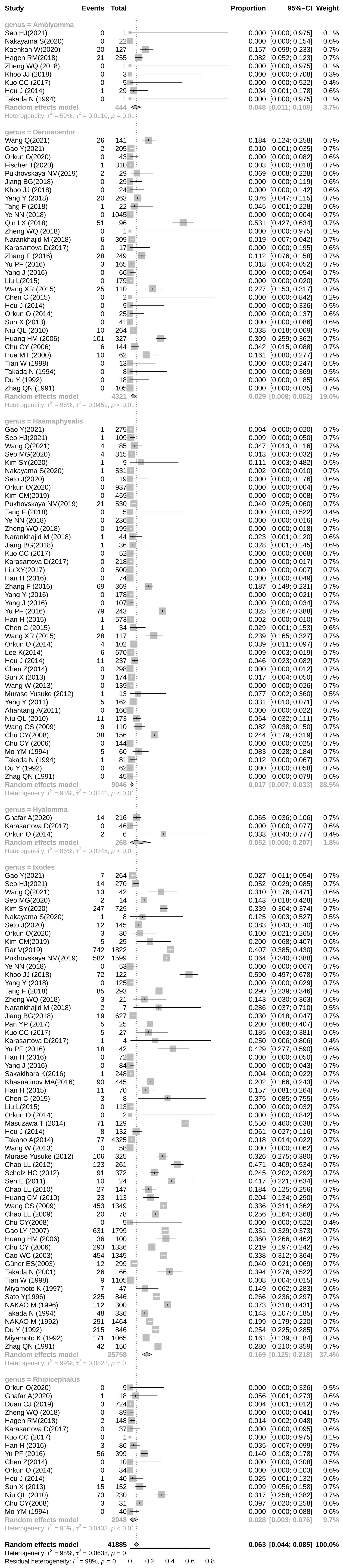

Study Events Total Proportion 95%−CI Weight
genus = Amblyomma
Seo HJ(2021) 0 1 0.000 [0.000; 0.975] 0.1%
Nakayama S(2020) 0 22 0.000 [0.000; 0.154] 0.6%
Kaenkan W(2020) 20 127 0.157 [0.099; 0.233] 0.7%
Hagen RM(2018) 21 255 0.082 [0.052; 0.123] 0.7%
Zheng WQ (2018) 0 1 0.000 [0.000; 0.975] 0.1%
Khoo JJ (2018) 0 3 0.000 [0.000; 0.708] 0.3%
Kuo CC (2017) 0 5 0.000 [0.000; 0.522] 0.4%
Hou J (2014) 1 29 0.034 [0.001; 0.178] 0.6%
Takada N (1994) 0 1 0.000 [0.000; 0.975] 0.1%
Random effects model 444 0.048 [0.011; 0.108] 3.7%
Heterogeneity: I^2 = 59%, τ^2 = 0.0110, p = 0.01
genus = Dermacentor
Wang Q(2021) 26 141 0.184 [0.124; 0.258] 0.7%
Gao Y(2021) 2 205 0.010 [0.001; 0.035] 0.7%
Orkun O(2020) 0 43 0.000 [0.000; 0.082] 0.6%
Fischer T(2020) 1 310 0.003 [0.000; 0.018] 0.7%
Pukhovskaya NM(2019) 2 29 0.069 [0.008; 0.228] 0.6%
Jiang BG(2018) 0 29 0.000 [0.000; 0.119] 0.6%
Khoo JJ (2018) 0 24 0.000 [0.000; 0.142] 0.6%
Yang Y (2018) 20 263 0.076 [0.047; 0.115] 0.7%
Tang F (2018) 1 22 0.045 [0.001; 0.228] 0.6%
Ye NN (2018) 0 1045 0.000 [0.000; 0.004] 0.7%
Qin LX (2018) 51 96 0.531 [0.427; 0.634] 0.7%
Zheng WQ (2018) 0 1 0.000 [0.000; 0.975] 0.1%
Narankhajid M (2018) 6 309 0.019 [0.007; 0.042] 0.7%
Karasartova D(2017) 0 17 0.000 [0.000; 0.195] 0.6%
Zhang F (2016) 28 249 0.112 [0.076; 0.158] 0.7%
Yu PF (2016) 3 165 0.018 [0.004; 0.052] 0.7%
Yang J (2016) 0 66 0.000 [0.000; 0.054] 0.7%
Liu L(2015) 0 179 0.000 [0.000; 0.020] 0.7%
Wang XR (2015) 25 110 0.227 [0.153; 0.317] 0.7%
Chen C (2015) 0 2 0.000 [0.000; 0.842] 0.2%
Hou J (2014) 0 9 0.000 [0.000; 0.336] 0.5%
Orkun O (2014) 0 25 0.000 [0.000; 0.137] 0.6%
Sun X (2013) 0 41 0.000 [0.000; 0.086] 0.6%
Niu QL (2010) 10 264 0.038 [0.018; 0.069] 0.7%
Huang HM (2006) 101 327 0.309 [0.259; 0.362] 0.7%
Chu CY (2006) 6 144 0.042 [0.015; 0.088] 0.7%
Hua MT (2000) 10 62 0.161 [0.080; 0.277] 0.7%
Tian W (1998) 0 13 0.000 [0.000; 0.247] 0.5%
Takada N (1994) 0 8 0.000 [0.000; 0.369] 0.5%
Du Y (1992) 0 18 0.000 [0.000; 0.185] 0.6%
Zhag QN (1991) 0 105 0.000 [0.000; 0.035] 0.7%
Random effects model 4321 0.029 [0.008; 0.062] 19.0%
Heterogeneity: I^2 = 96%, τ^2 = 0.0459, p < 0.01
genus = Haemaphysalis
Gao Y(2021) 1 275 0.004 [0.000; 0.020] 0.7%
Seo HJ(2021) 1 109 0.009 [0.000; 0.050] 0.7%
Wang Q(2021) 4 85 0.047 [0.013; 0.116] 0.7%
Seo MG(2020) 4 315 0.013 [0.003; 0.032] 0.7%
Kim SY(2020) 1 9 0.111 [0.003; 0.482] 0.5%
Nakayama S(2020) 1 531 0.002 [0.000; 0.010] 0.7%
Seto J(2020) 0 19 0.000 [0.000; 0.176] 0.6%
Orkun O(2020) 0 937 0.000 [0.000; 0.004] 0.7%
Kim CM(2019) 0 459 0.000 [0.000; 0.008] 0.7%
Pukhovskaya NM(2019) 21 530 0.040 [0.025; 0.060] 0.7%
Tang F (2018) 0 5 0.000 [0.000; 0.522] 0.4%
Ye NN (2018) 0 236 0.000 [0.000; 0.016] 0.7%
Zheng WQ (2018) 0 199 0.000 [0.000; 0.018] 0.7%
Narankhajid M (2018) 1 44 0.023 [0.001; 0.120] 0.6%
Jiang BG(2018) 1 36 0.028 [0.001; 0.145] 0.6%
Kuo CC (2017) 0 52 0.000 [0.000; 0.068] 0.7%
Karasartova D(2017) 0 218 0.000 [0.000; 0.017] 0.7%
Liu XY(2017) 0 500 0.000 [0.000; 0.007] 0.7%
Han H (2016) 0 74 0.000 [0.000; 0.049] 0.7%
Zhang F (2016) 69 369 0.187 [0.149; 0.231] 0.7%
Yang Y (2016) 0 178 0.000 [0.000; 0.021] 0.7%
Yang J (2016) 0 107 0.000 [0.000; 0.034] 0.7%
Yu PF (2016) 79 243 0.325 [0.267; 0.388] 0.7%
Han H (2015) 1 573 0.002 [0.000; 0.010] 0.7%
Chen C (2015) 1 34 0.029 [0.001; 0.153] 0.6%
Wang XR (2015) 28 117 0.239 [0.165; 0.327] 0.7%
Orkun O (2014) 4 102 0.039 [0.011; 0.097] 0.7%
Lee K(2014) 6 670 0.009 [0.003; 0.019] 0.7%
Hou J (2014) 11 237 0.046 [0.023; 0.082] 0.7%
Chen Z(2014) 0 298 0.000 [0.000; 0.012] 0.7%
Sun X (2013) 3 174 0.017 [0.004; 0.050] 0.7%
Wang W (2013) 0 139 0.000 [0.000; 0.026] 0.7%
Murase Yusuke (2012) 1 13 0.077 [0.002; 0.360] 0.5%
Yang Y (2011) 5 162 0.031 [0.010; 0.071] 0.7%
Ahantarig A(2011) 0 166 0.000 [0.000; 0.022] 0.7%
Niu QL (2010) 11 173 0.064 [0.032; 0.111] 0.7%
Wang CS (2009) 9 110 0.082 [0.038; 0.150] 0.7%
Chu CY(2008) 38 156 0.244 [0.179; 0.319] 0.7%
Chu CY (2006) 0 144 0.000 [0.000; 0.025] 0.7%
Mo YM (1994) 5 60 0.083 [0.028; 0.184] 0.7%
Takada N (1994) 1 81 0.012 [0.000; 0.067] 0.7%
Du Y (1992) 0 62 0.000 [0.000; 0.058] 0.7%
Zhag QN (1991) 0 45 0.000 [0.000; 0.079] 0.6%
Random effects model 9046 0.017 [0.007; 0.033] 28.5%
Heterogeneity: I^2 = 95%, τ^2 = 0.0241, p < 0.01
genus = Hyalomma
Ghafar A(2020) 14 216 0.065 [0.036; 0.106] 0.7%
Karasartova D(2017) 0 46 0.000 [0.000; 0.077] 0.6%
Orkun O (2014) 2 6 0.333 [0.043; 0.777] 0.4%
Random effects model 268 0.052 [0.000; 0.207] 1.8%
Heterogeneity: I^2 = 86%, τ^2 = 0.0345, p < 0.01
genus = Ixodes
Gao Y(2021) 7 264 0.027 [0.011; 0.054] 0.7%
Seo HJ(2021) 14 270 0.052 [0.029; 0.085] 0.7%
Wang Q(2021) 13 42 0.310 [0.176; 0.471] 0.6%
Seo MG(2020) 2 14 0.143 [0.018; 0.428] 0.5%
Kim SY(2020) 247 729 0.339 [0.304; 0.374] 0.7%
Nakayama S(2020) 1 8 0.125 [0.003; 0.527] 0.5%
Seto J(2020) 12 145 0.083 [0.043; 0.140] 0.7%
Orkun O(2020) 3 30 0.100 [0.021; 0.265] 0.6%
Kim CM(2019) 5 25 0.200 [0.068; 0.407] 0.6%
Rar V(2019) 742 1822 0.407 [0.385; 0.430] 0.7%
Pukhovskaya NM(2019) 582 1599 0.364 [0.340; 0.388] 0.7%
Ye NN (2018) 0 53 0.000 [0.000; 0.067] 0.7%
Khoo JJ (2018) 72 122 0.590 [0.497; 0.678] 0.7%
Yang Y (2018) 0 125 0.000 [0.000; 0.029] 0.7%
Tang F (2018) 85 293 0.290 [0.239; 0.346] 0.7%
Zheng WQ (2018) 3 21 0.143 [0.030; 0.363] 0.6%
Narankhajid M (2018) 2 7 0.286 [0.037; 0.710] 0.5%
Jiang BG(2018) 19 627 0.030 [0.018; 0.047] 0.7%
Pan YP (2017) 5 25 0.200 [0.068; 0.407] 0.6%
Kuo CC (2017) 5 27 0.185 [0.063; 0.381] 0.6%
Karasartova D(2017) 1 4 0.250 [0.006; 0.806] 0.4%
Yu PF (2016) 18 42 0.429 [0.277; 0.590] 0.6%
Han H (2016) 0 72 0.000 [0.000; 0.050] 0.7%
Yang J (2016) 0 84 0.000 [0.000; 0.043] 0.7%
Sakakibara K(2016) 1 248 0.004 [0.000; 0.022] 0.7%
Khasnatinov MA(2016) 90 445 0.202 [0.166; 0.243] 0.7%
Han H (2015) 11 70 0.157 [0.081; 0.264] 0.7%
Chen C (2015) 3 8 0.375 [0.085; 0.755] 0.5%
Liu L(2015) 0 113 0.000 [0.000; 0.032] 0.7%
Orkun O (2014) 0 2 0.000 [0.000; 0.842] 0.2%
Masuzawa T (2014) 71 129 0.550 [0.460; 0.638] 0.7%
Hou J (2014) 8 132 0.061 [0.027; 0.116] 0.7%
Takano A(2014) 77 4325 0.018 [0.014; 0.022] 0.7%
Wang W (2013) 0 58 0.000 [0.000; 0.062] 0.7%
Murase Yusuke (2012) 106 325 0.326 [0.275; 0.380] 0.7%
Chao LL (2012) 123 261 0.471 [0.409; 0.534] 0.7%
Scholz HC (2012) 91 372 0.245 [0.202; 0.292] 0.7%
Sen E (2011) 10 24 0.417 [0.221; 0.634] 0.6%
Chao LL (2010) 27 147 0.184 [0.125; 0.256] 0.7%
Huang CM (2010) 23 113 0.204 [0.134; 0.290] 0.7%
Wang CS (2009) 453 1349 0.336 [0.311; 0.362] 0.7%
Chao LL (2009) 20 78 0.256 [0.164; 0.368] 0.7%
Chu CY(2008) 0 5 0.000 [0.000; 0.522] 0.4%
Gao LY (2007) 631 1799 0.351 [0.329; 0.373] 0.7%
Huang HM (2006) 36 100 0.360 [0.266; 0.462] 0.7%
Chu CY (2006) 293 1336 0.219 [0.197; 0.242] 0.7%
Cao WC (2003) 454 1345 0.338 [0.312; 0.364] 0.7%
Güner ES(2003) 12 299 0.040 [0.021; 0.069] 0.7%
Takada N (2001) 26 66 0.394 [0.276; 0.522] 0.7%
Tian W (1998) 9 1105 0.008 [0.004; 0.015] 0.7%
Miyamoto K (1997) 7 47 0.149 [0.062; 0.283] 0.6%
Sato Y(1996) 225 846 0.266 [0.236; 0.297] 0.7%
NAKAO M (1996) 112 300 0.373 [0.318; 0.431] 0.7%
Takada N (1994) 48 336 0.143 [0.107; 0.185] 0.7%
NAKAO M (1992) 291 1464 0.199 [0.179; 0.220] 0.7%
Du Y (1992) 215 846 0.254 [0.225; 0.285] 0.7%
Miyamoto K (1992) 171 1065 0.161 [0.139; 0.184] 0.7%
Zhag QN (1991) 42 150 0.280 [0.210; 0.359] 0.7%
Random effects model 25758 0.169 [0.125; 0.218] 37.4%
Heterogeneity: I^2 = 99%, τ^2 = 0.0523, p = 0
genus = Rhipicephalus
Orkun O(2020) 0 9 0.000 [0.000; 0.336] 0.5%
Ghafar A(2020) 1 18 0.056 [0.001; 0.273] 0.6%
Duan CJ (2019) 3 724 0.004 [0.001; 0.012] 0.7%
Zheng WQ (2018) 0 89 0.000 [0.000; 0.041] 0.7%
Hagen RM(2018) 2 148 0.014 [0.002; 0.048] 0.7%
Karasartova D(2017) 0 37 0.000 [0.000; 0.095] 0.6%
Kuo CC (2017) 0 1 0.000 [0.000; 0.975] 0.1%
Han H (2016) 3 86 0.035 [0.007; 0.099] 0.7%
Yu PF (2016) 56 399 0.140 [0.108; 0.178] 0.7%
Chen Z(2014) 0 10 0.000 [0.000; 0.308] 0.5%
Orkun O (2014) 0 34 0.000 [0.000; 0.103] 0.6%
Hou J (2014) 1 40 0.025 [0.001; 0.132] 0.6%
Sun X (2013) 15 152 0.099 [0.056; 0.158] 0.7%
Niu QL (2010) 73 230 0.317 [0.258; 0.382] 0.7%
Chu CY(2008) 3 31 0.097 [0.020; 0.258] 0.6%
Mo YM (1994) 0 40 0.000 [0.000; 0.088] 0.6%
Random effects model 2048 0.028 [0.003; 0.076] 9.7%
Heterogeneity: I^2 = 95%, τ^2 = 0.0433, p < 0.01
Random effects model 41885 0.063 [0.044; 0.085] 100.0%
Heterogeneity: I^2 = 98%, τ^2 = 0.0638, p = 0
Residual heterogeneity: I^2 = 98%, p = 0
0 0.2 0.4 0.6 0.8

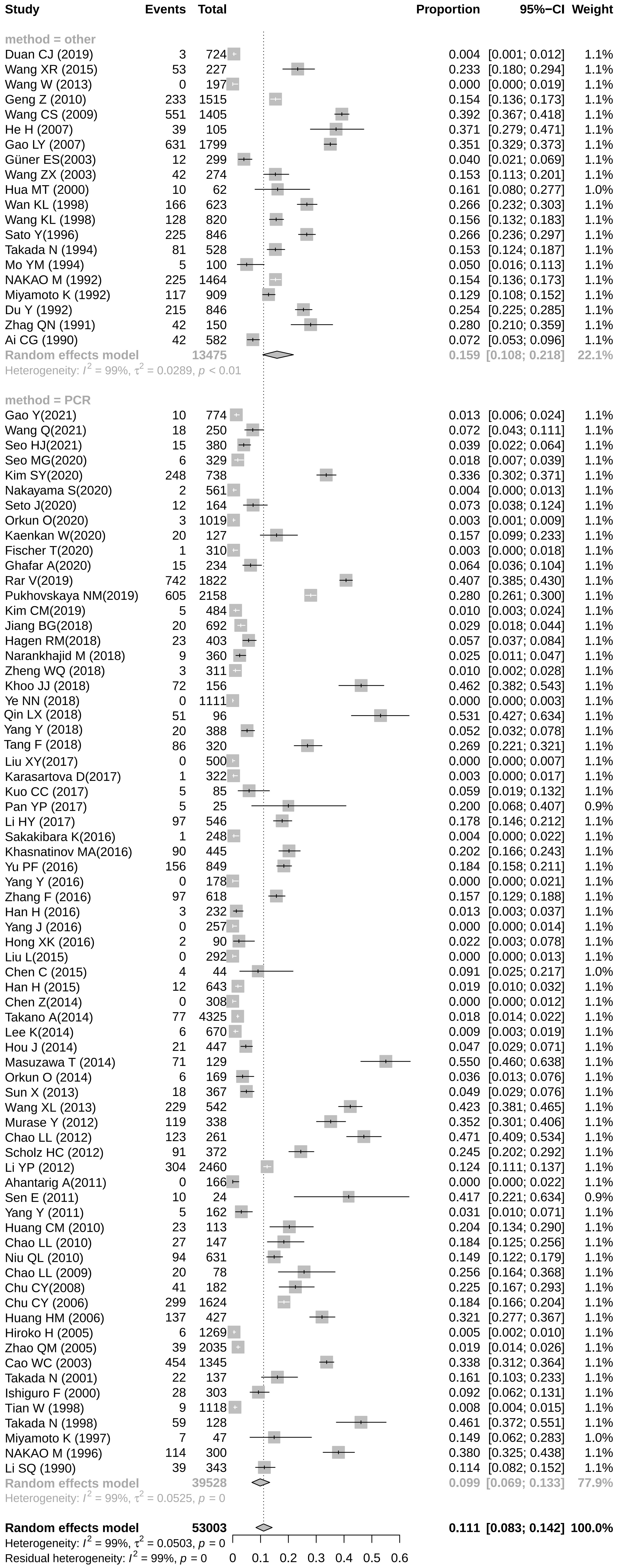
Study Events Total Proportion 95%−CI Weight
method = other
Duan CJ (2019) 3 724 0.004 [0.001; 0.012] 1.1%
Wang XR (2015) 53 227 0.233 [0.180; 0.294] 1.1%
Wang W (2013) 0 197 0.000 [0.000; 0.019] 1.1%
Geng Z (2010) 233 1515 0.154 [0.136; 0.173] 1.1%
Wang CS (2009) 551 1405 0.392 [0.367; 0.418] 1.1%
He H (2007) 39 105 0.371 [0.279; 0.471] 1.1%
Gao LY (2007) 631 1799 0.351 [0.329; 0.373] 1.1%
Güner ES(2003) 12 299 0.040 [0.021; 0.069] 1.1%
Wang ZX (2003) 42 274 0.153 [0.113; 0.201] 1.1%
Hua MT (2000) 10 62 0.161 [0.080; 0.277] 1.0%
Wan KL (1998) 166 623 0.266 [0.232; 0.303] 1.1%
Wang KL (1998) 128 820 0.156 [0.132; 0.183] 1.1%
Sato Y(1996) 225 846 0.266 [0.236; 0.297] 1.1%
Takada N (1994) 81 528 0.153 [0.124; 0.187] 1.1%
Mo YM (1994) 5 100 0.050 [0.016; 0.113] 1.1%
NAKAO M (1992) 225 1464 0.154 [0.136; 0.173] 1.1%
Miyamoto K (1992) 117 909 0.129 [0.108; 0.152] 1.1%
Du Y (1992) 215 846 0.254 [0.225; 0.285] 1.1%
Zhag QN (1991) 42 150 0.280 [0.210; 0.359] 1.1%
Ai CG (1990) 42 582 0.072 [0.053; 0.096] 1.1%
Random effects model 13475 0.159 [0.108; 0.218] 22.1%
Heterogeneity: I^2 = 99%, τ^2 = 0.0289, p < 0.01
method = PCR
Gao Y(2021) 10 774 0.013 [0.006; 0.024] 1.1%
Wang Q(2021) 18 250 0.072 [0.043; 0.111] 1.1%
Seo HJ(2021) 15 380 0.039 [0.022; 0.064] 1.1%
Seo MG(2020) 6 329 0.018 [0.007; 0.039] 1.1%
Kim SY(2020) 248 738 0.336 [0.302; 0.371] 1.1%
Nakayama S(2020) 2 561 0.004 [0.000; 0.013] 1.1%
Seto J(2020) 12 164 0.073 [0.038; 0.124] 1.1%
Orkun O(2020) 3 1019 0.003 [0.001; 0.009] 1.1%
Kaenkan W(2020) 20 127 0.157 [0.099; 0.233] 1.1%
Fischer T(2020) 1 310 0.003 [0.000; 0.018] 1.1%
Ghafar A(2020) 15 234 0.064 [0.036; 0.104] 1.1%
Rar V(2019) 742 1822 0.407 [0.385; 0.430] 1.1%
Pukhovskaya NM(2019) 605 2158 0.280 [0.261; 0.300] 1.1%
Kim CM(2019) 5 484 0.010 [0.003; 0.024] 1.1%
Jiang BG(2018) 20 692 0.029 [0.018; 0.044] 1.1%
Hagen RM(2018) 23 403 0.057 [0.037; 0.084] 1.1%
Narankhajid M (2018) 9 360 0.025 [0.011; 0.047] 1.1%
Zheng WQ (2018) 3 311 0.010 [0.002; 0.028] 1.1%
Khoo JJ (2018) 72 156 0.462 [0.382; 0.543] 1.1%
Ye NN (2018) 0 1111 0.000 [0.000; 0.003] 1.1%
Qin LX (2018) 51 96 0.531 [0.427; 0.634] 1.1%
Yang Y (2018) 20 388 0.052 [0.032; 0.078] 1.1%
Tang F (2018) 86 320 0.269 [0.221; 0.321] 1.1%
Liu XY(2017) 0 500 0.000 [0.000; 0.007] 1.1%
Karasartova D(2017) 1 322 0.003 [0.000; 0.017] 1.1%
Kuo CC (2017) 5 85 0.059 [0.019; 0.132] 1.1%
Pan YP (2017) 5 25 0.200 [0.068; 0.407] 0.9%
Li HY (2017) 97 546 0.178 [0.146; 0.212] 1.1%
Sakakibara K(2016) 1 248 0.004 [0.000; 0.022] 1.1%
Khasnatinov MA(2016) 90 445 0.202 [0.166; 0.243] 1.1%
Yu PF (2016) 156 849 0.184 [0.158; 0.211] 1.1%
Yang Y (2016) 0 178 0.000 [0.000; 0.021] 1.1%
Zhang F (2016) 97 618 0.157 [0.129; 0.188] 1.1%
Han H (2016) 3 232 0.013 [0.003; 0.037] 1.1%
Yang J (2016) 0 257 0.000 [0.000; 0.014] 1.1%
Hong XK (2016) 2 90 0.022 [0.003; 0.078] 1.1%
Liu L(2015) 0 292 0.000 [0.000; 0.013] 1.1%
Chen C (2015) 4 44 0.091 [0.025; 0.217] 1.0%
Han H (2015) 12 643 0.019 [0.010; 0.032] 1.1%
Chen Z(2014) 0 308 0.000 [0.000; 0.012] 1.1%
Takano A(2014) 77 4325 0.018 [0.014; 0.022] 1.1%
Lee K(2014) 6 670 0.009 [0.003; 0.019] 1.1%
Hou J (2014) 21 447 0.047 [0.029; 0.071] 1.1%
Masuzawa T (2014) 71 129 0.550 [0.460; 0.638] 1.1%
Orkun O (2014) 6 169 0.036 [0.013; 0.076] 1.1%
Sun X (2013) 18 367 0.049 [0.029; 0.076] 1.1%
Wang XL (2013) 229 542 0.423 [0.381; 0.465] 1.1%
Murase Y (2012) 119 338 0.352 [0.301; 0.406] 1.1%
Chao LL (2012) 123 261 0.471 [0.409; 0.534] 1.1%
Scholz HC (2012) 91 372 0.245 [0.202; 0.292] 1.1%
Li YP (2012) 304 2460 0.124 [0.111; 0.137] 1.1%
Ahantarig A(2011) 0 166 0.000 [0.000; 0.022] 1.1%
Sen E (2011) 10 24 0.417 [0.221; 0.634] 0.9%
Yang Y (2011) 5 162 0.031 [0.010; 0.071] 1.1%
Huang CM (2010) 23 113 0.204 [0.134; 0.290] 1.1%
Chao LL (2010) 27 147 0.184 [0.125; 0.256] 1.1%
Niu QL (2010) 94 631 0.149 [0.122; 0.179] 1.1%
Chao LL (2009) 20 78 0.256 [0.164; 0.368] 1.1%
Chu CY(2008) 41 182 0.225 [0.167; 0.293] 1.1%
Chu CY (2006) 299 1624 0.184 [0.166; 0.204] 1.1%
Huang HM (2006) 137 427 0.321 [0.277; 0.367] 1.1%
Hiroko H (2005) 6 1269 0.005 [0.002; 0.010] 1.1%
Zhao QM (2005) 39 2035 0.019 [0.014; 0.026] 1.1%
Cao WC (2003) 454 1345 0.338 [0.312; 0.364] 1.1%
Takada N (2001) 22 137 0.161 [0.103; 0.233] 1.1%
Ishiguro F (2000) 28 303 0.092 [0.062; 0.131] 1.1%
Tian W (1998) 9 1118 0.008 [0.004; 0.015] 1.1%
Takada N (1998) 59 128 0.461 [0.372; 0.551] 1.1%
Miyamoto K (1997) 7 47 0.149 [0.062; 0.283] 1.0%
NAKAO M (1996) 114 300 0.380 [0.325; 0.438] 1.1%
Li SQ (1990) 39 343 0.114 [0.082; 0.152] 1.1%
Random effects model 39528 0.099 [0.069; 0.133] 77.9%
Heterogeneity: I^2 = 99%, τ^2 = 0.0525, p = 0
Random effects model 53003 0.111 [0.083; 0.142] 100.0%
Heterogeneity: I^2 = 99%, τ^2 = 0.0503, p = 0
Residual heterogeneity: I^2 = 99%, p = 0
0 0.1 0.2 0.3 0.4 0.5 0.6

Study	Events	Total	Proportion	95%−CI	Weight
**periods = A**					
Gao Y(2021)	10	774	0.013	[0.006; 0.024]	1.1%
Wang Q(2021)	18	250	0.072	[0.043; 0.111]	1.1%
Seo HJ(2021)	15	380	0.039	[0.022; 0.064]	1.1%
Seo MG(2020)	6	329	0.018	[0.007; 0.039]	1.1%
Kim SY(2020)	248	738	0.336	[0.302; 0.371]	1.1%
Nakayama S(2020)	2	561	0.004	[0.000; 0.013]	1.1%
Seto J(2020)	12	164	0.073	[0.038; 0.124]	1.1%
Orkun O(2020)	3	1019	0.003	[0.001; 0.009]	1.1%
Kaenkan W(2020)	20	127	0.157	[0.099; 0.233]	1.1%
Fischer T(2020)	1	310	0.003	[0.000; 0.018]	1.1%
Ghafar A(2020)	15	234	0.064	[0.036; 0.104]	1.1%
Rar V(2019)	742	1822	0.407	[0.385; 0.430]	1.1%
Pukhovskaya NM(2019)	605	2158	0.280	[0.261; 0.300]	1.1%
Kim CM(2019)	5	484	0.010	[0.003; 0.024]	1.1%
Duan CJ (2019)	3	724	0.004	[0.001; 0.012]	1.1%
Jiang BG(2018)	20	692	0.029	[0.018; 0.044]	1.1%
Hagen RM(2018)	23	403	0.057	[0.037; 0.084]	1.1%
Narankhajid M (2018)	9	360	0.025	[0.011; 0.047]	1.1%
Zheng WQ (2018)	3	311	0.010	[0.002; 0.028]	1.1%
Khoo JJ (2018)	72	156	0.462	[0.382; 0.543]	1.1%
Ye NN (2018)	0	1111	0.000	[0.000; 0.003]	1.1%
Qin LX (2018)	51	96	0.531	[0.427; 0.634]	1.1%
Yang Y (2018)	20	388	0.052	[0.032; 0.078]	1.1%
Tang F (2018)	86	320	0.269	[0.221; 0.321]	1.1%
Liu XY(2017)	0	500	0.000	[0.000; 0.007]	1.1%
Karasartova D(2017)	1	322	0.003	[0.000; 0.017]	1.1%
Kuo CC (2017)	5	85	0.059	[0.019; 0.132]	1.1%
Pan YP (2017)	5	25	0.200	[0.068; 0.407]	0.9%
Li HY (2017)	97	546	0.178	[0.146; 0.212]	1.1%
Sakakibara K(2016)	1	248	0.004	[0.000; 0.022]	1.1%
Khasnatinov MA(2016)	90	445	0.202	[0.166; 0.243]	1.1%
Yu PF (2016)	156	849	0.184	[0.158; 0.211]	1.1%
Yang Y (2016)	0	178	0.000	[0.000; 0.021]	1.1%
Zhang F (2016)	97	618	0.157	[0.129; 0.188]	1.1%
Han H (2016)	3	232	0.013	[0.003; 0.037]	1.1%
Yang J (2016)	0	257	0.000	[0.000; 0.014]	1.1%
Hong XK (2016)	2	90	0.022	[0.003; 0.078]	1.1%
Liu L(2015)	0	292	0.000	[0.000; 0.013]	1.1%
Chen C (2015)	4	44	0.091	[0.025; 0.217]	1.0%
Han H (2015)	12	643	0.019	[0.010; 0.032]	1.1%
Wang XR (2015)	53	227	0.233	[0.180; 0.294]	1.1%
Chen Z(2014)	0	308	0.000	[0.000; 0.012]	1.1%
Takano A(2014)	77	4325	0.018	[0.014; 0.022]	1.1%
Lee K(2014)	6	670	0.009	[0.003; 0.019]	1.1%
Hou J (2014)	21	447	0.047	[0.029; 0.071]	1.1%
Masuzawa T (2014)	71	129	0.550	[0.460; 0.638]	1.1%
Orkun O (2014)	6	169	0.036	[0.013; 0.076]	1.1%
Wang W (2013)	0	197	0.000	[0.000; 0.019]	1.1%
Sun X (2013)	18	367	0.049	[0.029; 0.076]	1.1%
Wang XL (2013)	229	542	0.423	[0.381; 0.465]	1.1%
Murase Y (2012)	119	338	0.352	[0.301; 0.406]	1.1%
Chao LL (2012)	123	261	0.471	[0.409; 0.534]	1.1%
Scholz HC (2012)	91	372	0.245	[0.202; 0.292]	1.1%
Li YP (2012)	304	2460	0.124	[0.111; 0.137]	1.1%
Ahantarig A(2011)	0	166	0.000	[0.000; 0.022]	1.1%
Sen E (2011)	10	24	0.417	[0.221; 0.634]	0.9%
Yang Y (2011)	5	162	0.031	[0.010; 0.071]	1.1%
**Random effects model**		30449	0.078	[0.048; 0.114]	62.6%
Heterogeneity: $I^2 = 99\%$, $\tau^2 = 0.0549$, $p = 0$					
**periods = B**					
Huang CM (2010)	23	113	0.204	[0.134; 0.290]	1.1%
Chao LL (2010)	27	147	0.184	[0.125; 0.256]	1.1%
Geng Z (2010)	233	1515	0.154	[0.136; 0.173]	1.1%
Niu QL (2010)	94	631	0.149	[0.122; 0.179]	1.1%
Chao LL (2009)	20	78	0.256	[0.164; 0.368]	1.1%
Wang CS (2009)	551	1405	0.392	[0.367; 0.418]	1.1%
Chu CY(2008)	41	182	0.225	[0.167; 0.293]	1.1%
He H (2007)	39	105	0.371	[0.279; 0.471]	1.1%
Gao LY (2007)	631	1799	0.351	[0.329; 0.373]	1.1%
Chu CY (2006)	299	1624	0.184	[0.166; 0.204]	1.1%
Huang HM (2006)	137	427	0.321	[0.277; 0.367]	1.1%
Hiroko H (2005)	6	1269	0.005	[0.002; 0.010]	1.1%
Zhao QM (2005)	39	2035	0.019	[0.014; 0.026]	1.1%
Güner ES(2003)	12	299	0.040	[0.021; 0.069]	1.1%
Cao WC (2003)	454	1345	0.338	[0.312; 0.364]	1.1%
Wang ZX (2003)	42	274	0.153	[0.113; 0.201]	1.1%
Takada N (2001)	22	137	0.161	[0.103; 0.233]	1.1%
**Random effects model**		13385	0.186	[0.111; 0.274]	18.8%
Heterogeneity: $I^2 = 99\%$, $\tau^2 = 0.0483$, $p = 0$					
**periods = C**					
Ishiguro F (2000)	28	303	0.092	[0.062; 0.131]	1.1%
Hua MT (2000)	10	62	0.161	[0.080; 0.277]	1.0%
Tian W (1998)	9	1118	0.008	[0.004; 0.015]	1.1%
Takada N (1998)	59	128	0.461	[0.372; 0.551]	1.1%
Wan KL (1998)	166	623	0.266	[0.232; 0.303]	1.1%
Wang KL (1998)	128	820	0.156	[0.132; 0.183]	1.1%
Miyamoto K (1997)	7	47	0.149	[0.062; 0.283]	1.0%
Sato Y(1996)	225	846	0.266	[0.236; 0.297]	1.1%
NAKAO M (1996)	114	300	0.380	[0.325; 0.438]	1.1%
Takada N (1994)	81	528	0.153	[0.124; 0.187]	1.1%
Mo YM (1994)	5	100	0.050	[0.016; 0.113]	1.1%
NAKAO M (1992)	225	1464	0.154	[0.136; 0.173]	1.1%
Miyamoto K (1992)	117	909	0.129	[0.108; 0.152]	1.1%
Du Y (1992)	215	846	0.254	[0.225; 0.285]	1.1%
Zhag QN (1991)	42	150	0.280	[0.210; 0.359]	1.1%
Ai CG (1990)	42	582	0.072	[0.053; 0.096]	1.1%
Li SQ (1990)	39	343	0.114	[0.082; 0.152]	1.1%
**Random effects model**		9169	0.169	[0.116; 0.230]	18.7%
Heterogeneity: $I^2 = 98\%$, $\tau^2 = 0.0245$, $p < 0.01$					
**Random effects model**		**53003**	**0.111**	**[0.083; 0.142]**	**100.0%**
Heterogeneity: $I^2 = 99\%$, $\tau^2 = 0.0503$, $p = 0$					
Residual heterogeneity: $I^2 = 99\%$, $p = 0$					

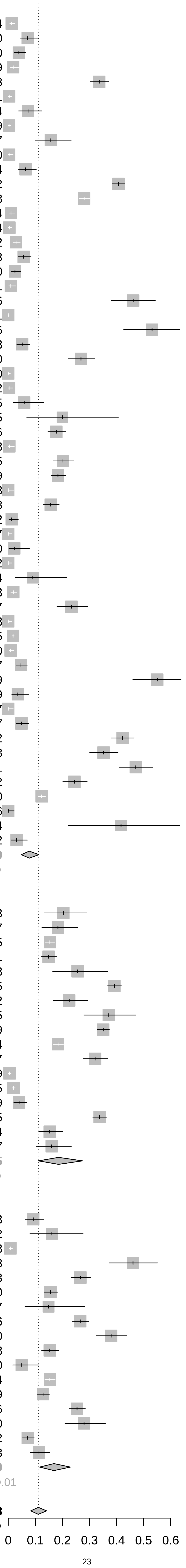

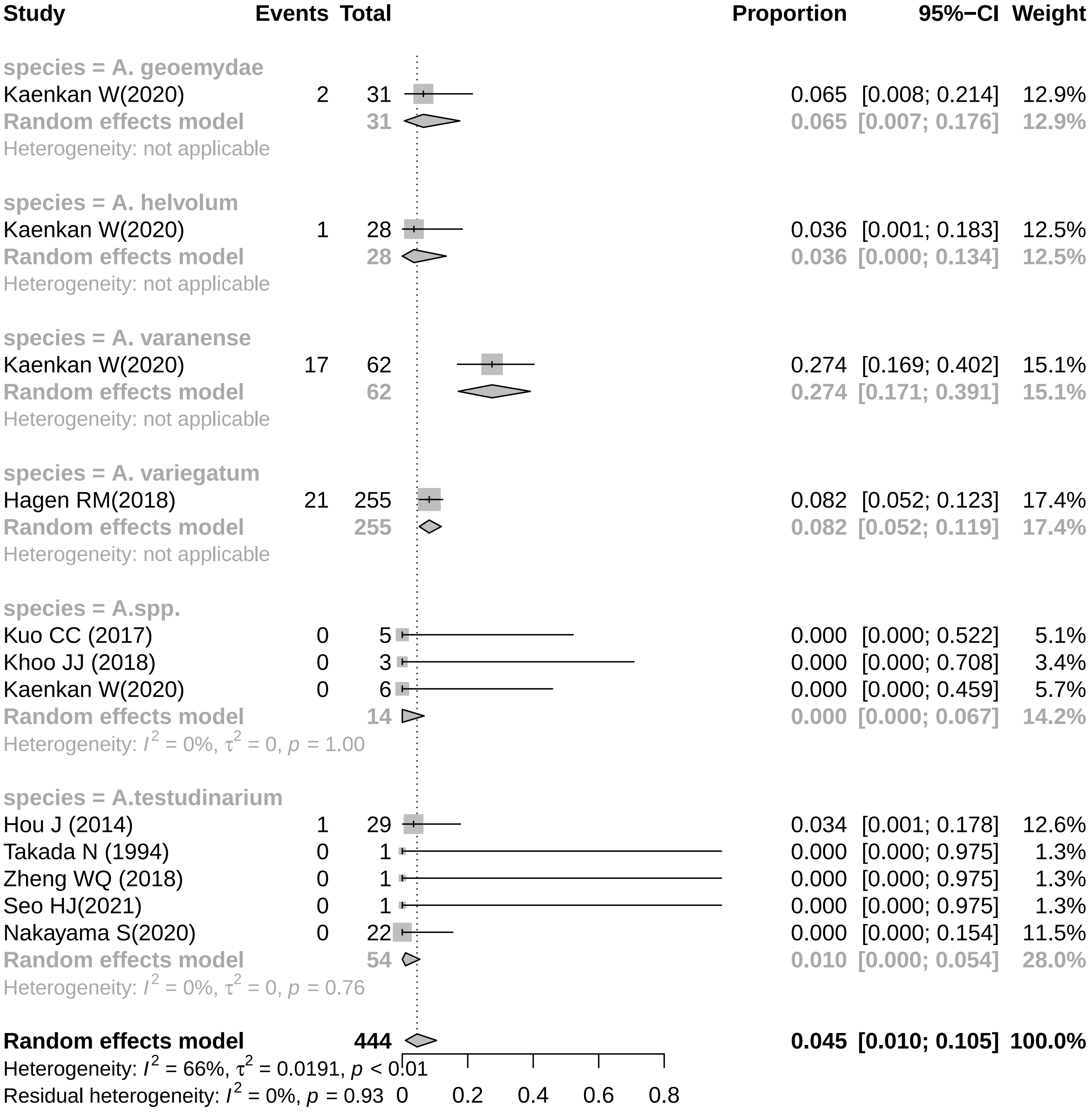
Study Events Total Proportion 95%−CI Weight
species = A. geoemydae
Kaenkan W(2020) 2 31 0.065 [0.008; 0.214] 12.9%
Random effects model 31 0.065 [0.007; 0.176] 12.9%
Heterogeneity: not applicable
species = A. helvolum
Kaenkan W(2020) 1 28 0.036 [0.001; 0.183] 12.5%
Random effects model 28 0.036 [0.000; 0.134] 12.5%
Heterogeneity: not applicable
species = A. varanense
Kaenkan W(2020) 17 62 0.274 [0.169; 0.402] 15.1%
Random effects model 62 0.274 [0.171; 0.391] 15.1%
Heterogeneity: not applicable
species = A. variegatum
Hagen RM(2018) 21 255 0.082 [0.052; 0.123] 17.4%
Random effects model 255 0.082 [0.052; 0.119] 17.4%
Heterogeneity: not applicable
species = A.spp.
Kuo CC (2017) 0 5 0.000 [0.000; 0.522] 5.1%
Khoo JJ (2018) 0 3 0.000 [0.000; 0.708] 3.4%
Kaenkan W(2020) 0 6 0.000 [0.000; 0.459] 5.7%
Random effects model 14 0.000 [0.000; 0.067] 14.2%
Heterogeneity: I^2 = 0%, τ^2 = 0, p = 1.00
species = A.testudinarium
Hou J (2014) 1 29 0.034 [0.001; 0.178] 12.6%
Takada N (1994) 0 1 0.000 [0.000; 0.975] 1.3%
Zheng WQ (2018) 0 1 0.000 [0.000; 0.975] 1.3%
Seo HJ(2021) 0 1 0.000 [0.000; 0.975] 1.3%
Nakayama S(2020) 0 22 0.000 [0.000; 0.154] 11.5%
Random effects model 54 0.010 [0.000; 0.054] 28.0%
Heterogeneity: I^2 = 0%, τ^2 = 0, p = 0.76
Random effects model 444 0.045 [0.010; 0.105] 100.0%
Heterogeneity: I^2 = 66%, τ^2 = 0.0191, p < 0.01
Residual heterogeneity: I^2 = 0%, p = 0.93
0 0.2 0.4 0.6 0.8

Study	Events	Total	Proportion	95%−CI	Weight
**species = D.auratus**					
Tian W (1998)	0	13	0.000	[0.000; 0.247]	2.4%
Narankhajid M (2018)	0	2	0.000	[0.000; 0.842]	0.9%
Yu PF (2016)	3	165	0.018	[0.004; 0.052]	3.2%
Chu CY (2006)	6	144	0.042	[0.015; 0.088]	3.2%
Hua MT (2000)	6	32	0.188	[0.072; 0.364]	2.8%
Ye NN (2018)	0	1045	0.000	[0.000; 0.004]	3.3%
Chen C (2015)	0	2	0.000	[0.000; 0.842]	0.9%
Du Y (1992)	0	18	0.000	[0.000; 0.185]	2.6%
Huang HM (2006)	101	327	0.309	[0.259; 0.362]	3.3%
Tang F (2018)	1	22	0.045	[0.001; 0.228]	2.7%
Yang J (2016)	0	66	0.000	[0.000; 0.054]	3.1%
**Random effects model**		**1836**	**0.027**	**[0.000; 0.116]**	**28.3%**
Heterogeneity: $I^2 = 97\%$, $\tau^2 = 0.0778$, $p < 0.01$					
**species = D.marginatus**					
Niu QL (2010)	10	264	0.038	[0.018; 0.069]	3.3%
Qin LX (2018)	51	96	0.531	[0.427; 0.634]	3.1%
Orkun O(2020)	0	41	0.000	[0.000; 0.086]	2.9%
Karasartova D(2017)	0	17	0.000	[0.000; 0.195]	2.5%
**Random effects model**		**418**	**0.066**	**[0.000; 0.359]**	**11.9%**
Heterogeneity: $I^2 = 98\%$, $\tau^2 = 0.1466$, $p < 0.01$					
**species = D.niveus**					
Khoo JJ (2018)	0	24	0.000	[0.000; 0.142]	2.7%
**Random effects model**		**24**	**0.000**	**[0.000; 0.039]**	**2.7%**
Heterogeneity: not applicable					
**species = D.nuttalli**					
Narankhajid M (2018)	0	35	0.000	[0.000; 0.100]	2.9%
Zhag QN (1991)	0	105	0.000	[0.000; 0.035]	3.2%
Orkun O (2014)	0	25	0.000	[0.000; 0.137]	2.7%
Sun X (2013)	0	41	0.000	[0.000; 0.086]	2.9%
Wang Q(2021)	0	21	0.000	[0.000; 0.161]	2.6%
Fischer T(2020)	1	310	0.003	[0.000; 0.018]	3.3%
Gao Y(2021)	1	127	0.008	[0.000; 0.043]	3.2%
Liu L(2015)	0	179	0.000	[0.000; 0.020]	3.2%
**Random effects model**		**843**	**0.001**	**[0.000; 0.005]**	**24.0%**
Heterogeneity: $I^2 = 0\%$, $\tau^2 = 0$, $p = 0.77$					
**species = D.silvarum**					
Hou J (2014)	0	9	0.000	[0.000; 0.336]	2.1%
Takada N (1994)	0	8	0.000	[0.000; 0.369]	2.0%
Wang Q(2021)	1	120	0.008	[0.000; 0.046]	3.2%
Pukhovskaya NM(2019)	2	29	0.069	[0.008; 0.228]	2.8%
Gao Y(2021)	1	78	0.013	[0.000; 0.069]	3.1%
Jiang BG(2018)	0	29	0.000	[0.000; 0.119]	2.8%
**Random effects model**		**273**	**0.010**	**[0.002; 0.026]**	**16.0%**
Heterogeneity: $I^2 = 2\%$, $\tau^2 = 0.0002$, $p = 0.40$					
**species = D.spp**					
Zheng WQ (2018)	0	1	0.000	[0.000; 0.975]	0.5%
Hua MT (2000)	4	30	0.133	[0.038; 0.307]	2.8%
Yang Y (2018)	45	263	0.171	[0.128; 0.222]	3.3%
Zhang F (2016)	28	249	0.112	[0.076; 0.158]	3.2%
Wang XR (2015)	25	110	0.227	[0.153; 0.317]	3.2%
Orkun O(2020)	0	2	0.000	[0.000; 0.842]	0.9%
**Random effects model**		**655**	**0.155**	**[0.108; 0.208]**	**13.9%**
Heterogeneity: $I^2 = 51\%$, $\tau^2 = 0.0030$, $p = 0.07$					
**species = D.taiwanensis**					
Narankhajid M (2018)	6	272	0.022	[0.008; 0.047]	3.3%
**Random effects model**		**272**	**0.022**	**[0.008; 0.043]**	**3.3%**
Heterogeneity: not applicable					
**Random effects model**		**4321**	**0.024**	**[0.006; 0.053]**	**100.0%**
Heterogeneity: $I^2 = 95\%$, $\tau^2 = 0.0470$, $p < 0.01$					
Residual heterogeneity: $I^2 = 94\%$, $p < 0.01$					

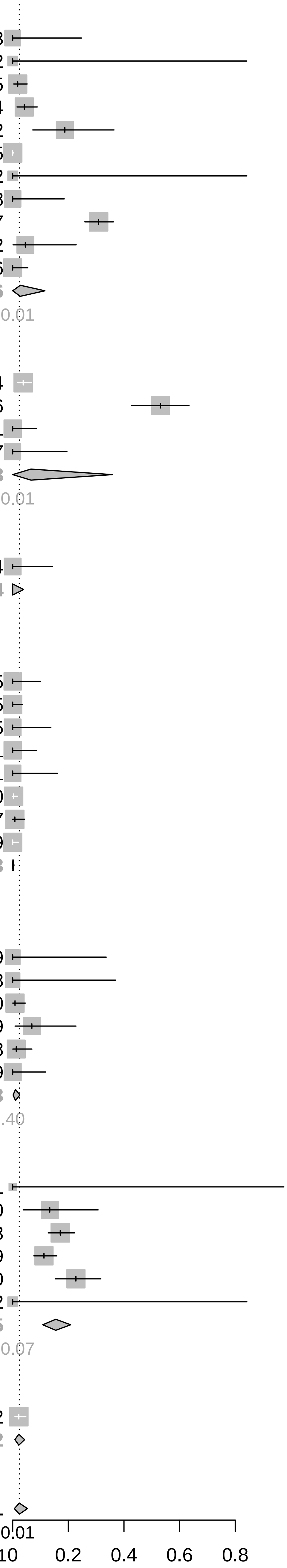

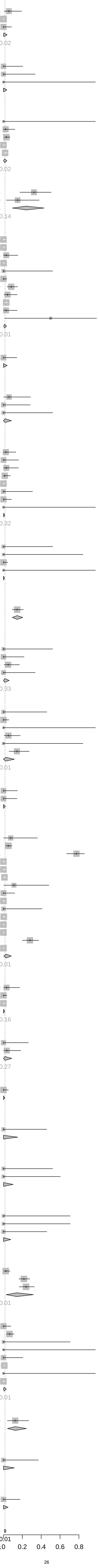

Study	Events	Total	Proportion	95%−CI	Weight
**species = H. parva**					
Orkun O (2014)	2	35	0.057	[0.007; 0.192]	1.2%
Orkun O(2020)	0	793	0.000	[0.000; 0.005]	1.6%
Karasartova D(2017)	0	41	0.000	[0.000; 0.086]	1.3%
**Random effects model**		**869**	**0.004**	**[0.000; 0.039]**	**4.1%**
Heterogeneity: $I^2 = 74\%$, $\tau^2 = 0.0103$, $p = 0.02$					
**species = H.aegyptium**					
Orkun O (2014)	0	16	0.000	[0.000; 0.206]	1.0%
Orkun O(2020)	0	9	0.000	[0.000; 0.336]	0.8%
Karasartova D(2017)	0	1	0.000	[0.000; 0.975]	0.2%
**Random effects model**		**26**	**0.000**	**[0.000; 0.036]**	**1.9%**
Heterogeneity: $I^2 = 0\%$, $\tau^2 = 0$, $p = 1.00$					
**species = H.asiaticum**					
Hou J (2014)	0	1	0.000	[0.000; 0.975]	0.2%
Narankhajid M (2018)	1	44	0.023	[0.001; 0.120]	1.3%
Yang Y (2011)	5	162	0.031	[0.010; 0.071]	1.5%
Yang Y (2016)	0	178	0.000	[0.000; 0.021]	1.5%
Sun X (2013)	3	174	0.017	[0.004; 0.050]	1.5%
**Random effects model**		**559**	**0.012**	**[0.001; 0.037]**	**5.9%**
Heterogeneity: $I^2 = 67\%$, $\tau^2 = 0.0051$, $p = 0.02$					
**species = H.bispinosa**					
Yu PF (2016)	11	34	0.324	[0.174; 0.505]	1.2%
Mo YM (1994)	3	20	0.150	[0.032; 0.379]	1.1%
**Random effects model**		**54**	**0.242**	**[0.094; 0.430]**	**2.3%**
Heterogeneity: $I^2 = 54\%$, $\tau^2 = 0.0116$, $p = 0.14$					
**species = H.concinna**					
Chu CY (2006)	0	144	0.000	[0.000; 0.025]	1.5%
Ye NN (2018)	0	236	0.000	[0.000; 0.016]	1.5%
Chen C (2015)	1	34	0.029	[0.001; 0.153]	1.2%
Han H (2015)	1	573	0.002	[0.000; 0.010]	1.5%
Tang F (2018)	0	5	0.000	[0.000; 0.522]	0.5%
Yang J (2016)	0	106	0.000	[0.000; 0.034]	1.4%
Wang CS (2009)	9	110	0.082	[0.038; 0.150]	1.4%
Wang Q(2021)	2	47	0.043	[0.005; 0.145]	1.3%
Pukhovskaya NM(2019)	14	481	0.029	[0.016; 0.048]	1.5%
Jiang BG(2018)	1	36	0.028	[0.001; 0.145]	1.2%
Chu CY(2008)	1	2	0.500	[0.013; 0.987]	0.3%
**Random effects model**		**1774**	**0.013**	**[0.002; 0.032]**	**13.6%**
Heterogeneity: $I^2 = 83\%$, $\tau^2 = 0.0089$, $p < 0.01$					
**species = H.doenitzi**					
Kuo CC (2017)	0	24	0.000	[0.000; 0.142]	1.1%
**Random effects model**		**24**	**0.000**	**[0.000; 0.039]**	**1.1%**
Heterogeneity: not applicable					
**species = H.excavatum**					
Orkun O (2014)	1	17	0.059	[0.001; 0.287]	1.0%
Orkun O(2020)	0	11	0.000	[0.000; 0.285]	0.8%
Karasartova D(2017)	0	5	0.000	[0.000; 0.522]	0.5%
**Random effects model**		**33**	**0.016**	**[0.000; 0.086]**	**2.4%**
Heterogeneity: $I^2 = 0\%$, $\tau^2 = 0$, $p = 0.37$					
**species = H.flava**					
Takada N (1994)	1	40	0.025	[0.001; 0.132]	1.3%
Zheng WQ (2018)	0	21	0.000	[0.000; 0.161]	1.1%
Seo MG(2020)	1	33	0.030	[0.001; 0.158]	1.2%
Seo HJ(2021)	1	74	0.014	[0.000; 0.073]	1.4%
Nakayama S(2020)	0	213	0.000	[0.000; 0.017]	1.5%
Seto J(2020)	0	10	0.000	[0.000; 0.308]	0.8%
Kim CM(2019)	0	42	0.000	[0.000; 0.084]	1.3%
Kuo CC (2017)	0	1	0.000	[0.000; 0.975]	0.2%
**Random effects model**		**434**	**0.003**	**[0.000; 0.013]**	**8.7%**
Heterogeneity: $I^2 = 14\%$, $\tau^2 = 0.0009$, $p = 0.32$					
**species = H.formosensis**					
Han H (2016)	0	5	0.000	[0.000; 0.522]	0.5%
Seo HJ(2021)	0	2	0.000	[0.000; 0.842]	0.3%
Nakayama S(2020)	0	78	0.000	[0.000; 0.046]	1.4%
Kuo CC (2017)	0	1	0.000	[0.000; 0.975]	0.2%
**Random effects model**		**86**	**0.000**	**[0.000; 0.011]**	**2.4%**
Heterogeneity: $I^2 = 0\%$, $\tau^2 = 0$, $p = 1.00$					
**species = H.garhwalensis**					
Zhang F (2016)	22	152	0.145	[0.093; 0.211]	1.5%
**Random effects model**		**152**	**0.145**	**[0.094; 0.205]**	**1.5%**
Heterogeneity: not applicable					
**species = H.hystricis**					
Hou J (2014)	0	5	0.000	[0.000; 0.522]	0.5%
Nakayama S(2020)	0	15	0.000	[0.000; 0.218]	1.0%
Mo YM (1994)	2	40	0.050	[0.006; 0.169]	1.3%
Kuo CC (2017)	0	9	0.000	[0.000; 0.336]	0.8%
**Random effects model**		**69**	**0.014**	**[0.000; 0.061]**	**3.5%**
Heterogeneity: $I^2 = 12\%$, $\tau^2 = 0.0026$, $p = 0.33$					
**species = H.japonica**					
Takada N (1994)	0	6	0.000	[0.000; 0.459]	0.6%
Du Y (1992)	0	62	0.000	[0.000; 0.058]	1.4%
Yang J (2016)	0	1	0.000	[0.000; 0.975]	0.2%
Wang Q(2021)	2	38	0.053	[0.006; 0.177]	1.3%
Seto J(2020)	0	2	0.000	[0.000; 0.842]	0.3%
Pukhovskaya NM(2019)	7	49	0.143	[0.059; 0.272]	1.3%
**Random effects model**		**158**	**0.025**	**[0.000; 0.115]**	**5.0%**
Heterogeneity: $I^2 = 72\%$, $\tau^2 = 0.0299$, $p < 0.01$					
**species = H.kitaokai**					
Takada N (1994)	0	23	0.000	[0.000; 0.148]	1.1%
Nakayama S(2020)	0	24	0.000	[0.000; 0.142]	1.1%
**Random effects model**		**47**	**0.000**	**[0.000; 0.020]**	**2.2%**
Heterogeneity: $I^2 = 0\%$, $\tau^2 = 0$, $p = 1.00$					
**species = H.longicornis**					
Murase Yusuke (2012)	1	13	0.077	[0.002; 0.360]	0.9%
Hou J (2014)	11	212	0.052	[0.026; 0.091]	1.5%
Yu PF (2016)	65	84	0.774	[0.670; 0.858]	1.4%
Zheng WQ (2018)	0	173	0.000	[0.000; 0.021]	1.5%
Wang W (2013)	0	139	0.000	[0.000; 0.026]	1.5%
Seo MG(2020)	3	282	0.011	[0.002; 0.031]	1.5%
Kim SY(2020)	1	9	0.111	[0.003; 0.482]	0.8%
Seo HJ(2021)	0	29	0.000	[0.000; 0.119]	1.2%
Nakayama S(2020)	0	173	0.000	[0.000; 0.021]	1.5%
Seto J(2020)	0	7	0.000	[0.000; 0.410]	0.7%
Gao Y(2021)	1	275	0.004	[0.000; 0.020]	1.5%
Chen Z(2014)	0	298	0.000	[0.000; 0.012]	1.5%
Liu XY(2017)	0	500	0.000	[0.000; 0.007]	1.5%
Chu CY(2008)	32	114	0.281	[0.201; 0.373]	1.4%
Kim CM(2019)	0	417	0.000	[0.000; 0.009]	1.5%
**Random effects model**		**2725**	**0.030**	**[0.003; 0.084]**	**20.0%**
Heterogeneity: $I^2 = 97\%$, $\tau^2 = 0.0505$, $p < 0.01$					
**species = H.marginatum**					
Orkun O (2014)	1	30	0.033	[0.001; 0.172]	1.2%
Orkun O(2020)	0	105	0.000	[0.000; 0.035]	1.4%
Karasartova D(2017)	0	164	0.000	[0.000; 0.022]	1.5%
**Random effects model**		**299**	**0.001**	**[0.000; 0.013]**	**4.1%**
Heterogeneity: $I^2 = 45\%$, $\tau^2 = 0.0024$, $p = 0.16$					
**species = H.megaspinosa**					
Takada N (1994)	0	12	0.000	[0.000; 0.265]	0.9%
Nakayama S(2020)	1	28	0.036	[0.001; 0.183]	1.2%
**Random effects model**		**40**	**0.016**	**[0.000; 0.089]**	**2.1%**
Heterogeneity: $I^2 = 18\%$, $\tau^2 = 0.0032$, $p = 0.27$					
**species = H.nepalensis**					
Han H (2016)	0	69	0.000	[0.000; 0.052]	1.4%
**Random effects model**		**69**	**0.000**	**[0.000; 0.014]**	**1.4%**
Heterogeneity: not applicable					
**species = H.ornithophila**					
Kuo CC (2017)	0	6	0.000	[0.000; 0.459]	0.6%
**Random effects model**		**6**	**0.000**	**[0.000; 0.152]**	**0.6%**
Heterogeneity: not applicable					
**species = H.phasiana**					
Zheng WQ (2018)	0	5	0.000	[0.000; 0.522]	0.5%
Seo HJ(2021)	0	4	0.000	[0.000; 0.602]	0.5%
**Random effects model**		**9**	**0.000**	**[0.000; 0.103]**	**1.0%**
Heterogeneity: $I^2 = 0\%$, $\tau^2 = 0$, $p = 1.00$					
**species = H.punctata**					
Orkun O (2014)	0	3	0.000	[0.000; 0.708]	0.4%
Orkun O(2020)	0	3	0.000	[0.000; 0.708]	0.4%
Karasartova D(2017)	0	6	0.000	[0.000; 0.459]	0.6%
**Random effects model**		**12**	**0.000**	**[0.000; 0.078]**	**1.4%**
Heterogeneity: $I^2 = 0\%$, $\tau^2 = 0$, $p = 1.00$					
**species = H.qinghaiensis**					
Yu PF (2016)	3	125	0.024	[0.005; 0.069]	1.5%
Zhang F (2016)	47	217	0.217	[0.164; 0.277]	1.5%
Wang XR (2015)	28	117	0.239	[0.165; 0.327]	1.5%
**Random effects model**		**459**	**0.141**	**[0.029; 0.317]**	**4.4%**
Heterogeneity: $I^2 = 95\%$, $\tau^2 = 0.0340$, $p < 0.01$					
**species = H.spp**					
Zhag QN (1991)	0	45	0.000	[0.000; 0.079]	1.3%
Niu QL (2010)	11	173	0.064	[0.032; 0.111]	1.5%
Kuo CC (2017)	0	3	0.000	[0.000; 0.708]	0.4%
Orkun O (2014)	0	1	0.000	[0.000; 0.975]	0.2%
Orkun O(2020)	0	16	0.000	[0.000; 0.206]	1.0%
Lee K(2014)	6	670	0.009	[0.003; 0.019]	1.5%
Karasartova D(2017)	0	1	0.000	[0.000; 0.975]	0.2%
Ahantarig A(2011)	0	166	0.000	[0.000; 0.022]	1.5%
**Random effects model**		**1075**	**0.007**	**[0.000; 0.030]**	**7.5%**
Heterogeneity: $I^2 = 73\%$, $\tau^2 = 0.0079$, $p < 0.01$					
**species = H.warburtoni**					
Chu CY(2008)	5	40	0.125	[0.042; 0.268]	1.3%
**Random effects model**		**40**	**0.125**	**[0.042; 0.244]**	**1.3%**
Heterogeneity: not applicable					
**species = H.wellingtoni**					
Kuo CC (2017)	0	8	0.000	[0.000; 0.369]	0.7%
**Random effects model**		**8**	**0.000**	**[0.000; 0.115]**	**0.7%**
Heterogeneity: not applicable					
**species = H.yeni**					
Hou J (2014)	0	19	0.000	[0.000; 0.176]	1.0%
**Random effects model**		**19**	**0.000**	**[0.000; 0.050]**	**1.0%**
Heterogeneity: not applicable					
**Random effects model**		**9046**	**0.016**	**[0.008; 0.028]**	**100.0%**
Heterogeneity: $I^2 = 91\%$, $\tau^2 = 0.0265$, $p < 0.01$					
Residual heterogeneity: $I^2 = 90\%$, $p < 0.01$					

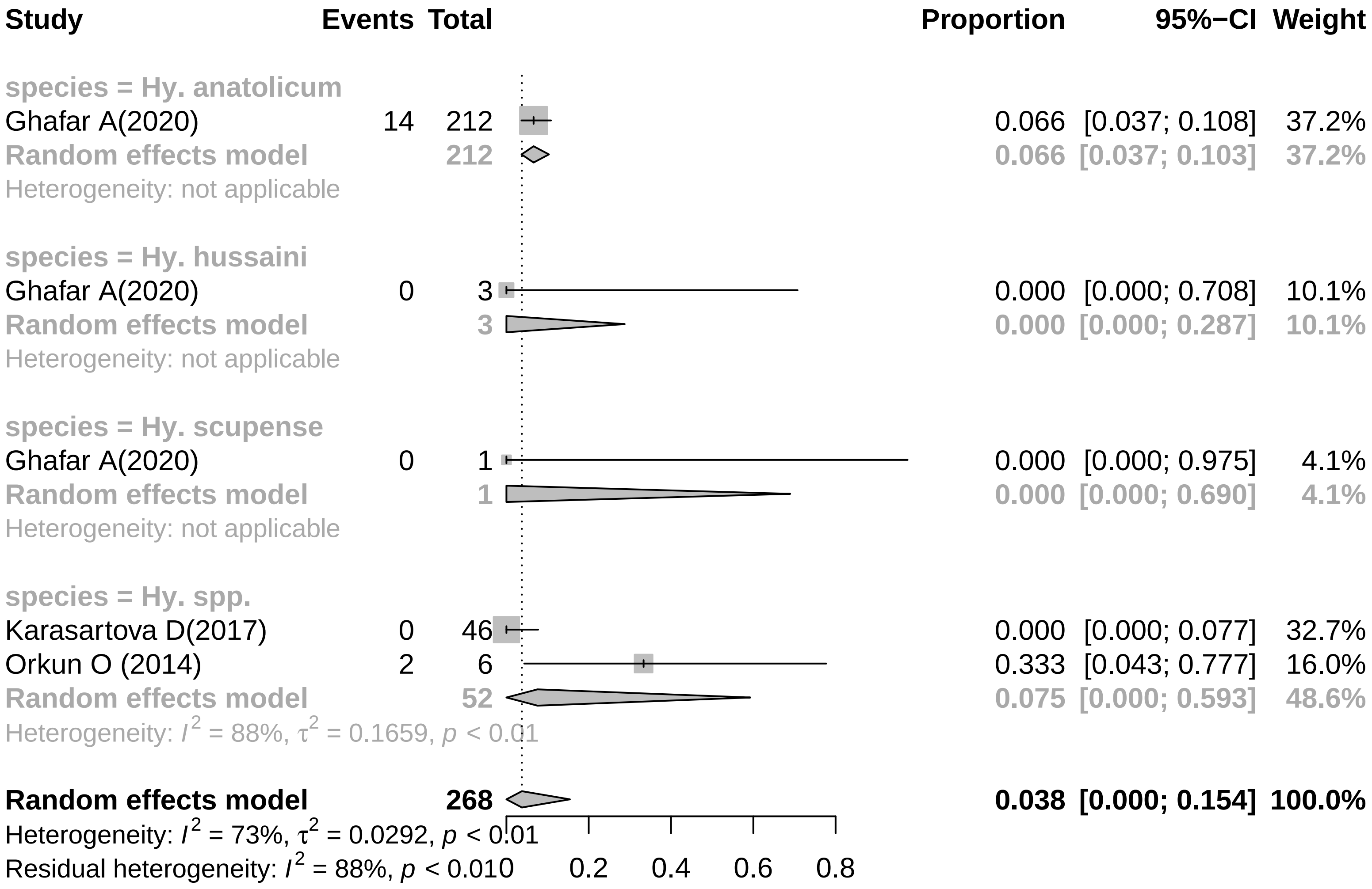

Study
Events
Total
Proportion
95%−CI
Weight
species = Hy. anatolicum
Ghafar A(2020) 14 212 0.066 [0.037; 0.108] 37.2%
Random effects model 212 0.066 [0.037; 0.103] 37.2%
Heterogeneity: not applicable
species = Hy. hussaini
Ghafar A(2020) 0 3 0.000 [0.000; 0.708] 10.1%
Random effects model 3 0.000 [0.000; 0.287] 10.1%
Heterogeneity: not applicable
species = Hy. scupense
Ghafar A(2020) 0 1 0.000 [0.000; 0.975] 4.1%
Random effects model 1 0.000 [0.000; 0.690] 4.1%
Heterogeneity: not applicable
species = Hy. spp.
Karasartova D(2017) 0 46 0.000 [0.000; 0.077] 32.7%
Orkun O (2014) 2 6 0.333 [0.043; 0.777] 16.0%
Random effects model 52 0.075 [0.000; 0.593] 48.6%
Heterogeneity: I^2 = 88%, τ^2 = 0.1659, p < 0.01
Random effects model 268 0.038 [0.000; 0.154] 100.0%
Heterogeneity: I^2 = 73%, τ^2 = 0.0292, p < 0.01
Residual heterogeneity: I^2 = 88%, p < 0.01
0
0.2
0.4
0.6
0.8

Study	Events	Total	Proportion	95%−CI	Weight
**species = I. ovatus**					
Murase Yusuke (2012)	8	41	0.195	[0.088; 0.349]	1.3%
Hou J (2014)	0	2	0.000	[0.000; 0.842]	0.4%
NAKAO M (1992)	162	687	0.236	[0.205; 0.269]	1.4%
Takada N (1994)	37	193	0.192	[0.139; 0.254]	1.4%
Miyamoto K (1992)	41	273	0.150	[0.110; 0.198]	1.4%
Nakayama S(2020)	0	5	0.000	[0.000; 0.522]	0.8%
Seto J(2020)	11	95	0.116	[0.059; 0.198]	1.4%
Takano A(2014)	1	676	0.001	[0.000; 0.008]	1.4%
Random effects model		1972	0.098	[0.019; 0.230]	9.5%
Heterogeneity: I^2 = 98%, τ^2 = 0.0573, p < 0.01					
**species = I. acuminatus**					
Zheng WQ (2018)	0	1	0.000	[0.000; 0.975]	0.3%
Random effects model		1	0.000	[0.000; 0.690]	0.3%
Heterogeneity: not applicable					
**species = I. angustus**					
Kim SY(2020)	3	96	0.031	[0.006; 0.089]	1.4%
Random effects model		96	0.031	[0.006; 0.075]	1.4%
Heterogeneity: not applicable					
**species = I. columnae**					
Kuo CC (2017)	0	17	0.000	[0.000; 0.195]	1.1%
Random effects model		17	0.000	[0.000; 0.055]	1.1%
Heterogeneity: not applicable					
**species = I. granulatus**					
Chao LL (2012)	123	261	0.471	[0.409; 0.534]	1.4%
Chao LL (2009)	20	78	0.256	[0.164; 0.368]	1.4%
Huang CM (2010)	23	113	0.204	[0.134; 0.290]	1.4%
Chao LL (2010)	27	147	0.184	[0.125; 0.256]	1.4%
Tian W (1998)	4	947	0.004	[0.001; 0.011]	1.4%
Hou J (2014)	7	36	0.194	[0.082; 0.360]	1.3%
Zheng WQ (2018)	3	16	0.188	[0.040; 0.456]	1.1%
Khoo JJ (2018)	72	122	0.590	[0.497; 0.678]	1.4%
Chu CY (2006)	293	1336	0.219	[0.197; 0.242]	1.4%
Random effects model		3056	0.233	[0.094; 0.411]	12.2%
Heterogeneity: I^2 = 99%, τ^2 = 0.0830, p < 0.01					
**species = I. monospinosus**					
Takada N (1994)	0	58	0.000	[0.000; 0.062]	1.3%
Random effects model		58	0.000	[0.000; 0.016]	1.3%
Heterogeneity: not applicable					
**species = I. nipponensis**					
Kuo CC (2017)	0	1	0.000	[0.000; 0.975]	0.3%
Seo MG(2020)	2	14	0.143	[0.018; 0.428]	1.1%
Kim SY(2020)	109	193	0.565	[0.492; 0.636]	1.4%
Seo HJ(2021)	0	40	0.000	[0.000; 0.088]	1.3%
Seto J(2020)	0	37	0.000	[0.000; 0.095]	1.3%
Kim CM(2019)	5	25	0.200	[0.068; 0.407]	1.2%
Random effects model		310	0.094	[0.000; 0.430]	6.5%
Heterogeneity: I^2 = 97%, τ^2 = 0.2249, p < 0.01					
**species = I. pavlovskyi**					
Takano A(2014)	5	117	0.043	[0.014; 0.097]	1.4%
Random effects model		117	0.043	[0.014; 0.087]	1.4%
Heterogeneity: not applicable					
**species = I. persulcatus**					
Murase Yusuke (2012)	98	284	0.345	[0.290; 0.404]	1.4%
Scholz HC (2012)	91	372	0.245	[0.202; 0.292]	1.4%
Takada N (2001)	26	66	0.394	[0.276; 0.522]	1.3%
Tian W (1998)	5	158	0.032	[0.010; 0.072]	1.4%
Miyamoto K (1997)	7	47	0.149	[0.062; 0.283]	1.3%
Cao WC (2003)	454	1345	0.338	[0.312; 0.364]	1.4%
Masuzawa T (2014)	71	129	0.550	[0.460; 0.638]	1.4%
Kuo CC (2017)	3	4	0.750	[0.194; 0.994]	0.7%
Narankhajid M (2018)	2	7	0.286	[0.037; 0.710]	0.9%
NAKAO M (1992)	129	777	0.166	[0.141; 0.194]	1.4%
NAKAO M (1996)	112	300	0.373	[0.318; 0.431]	1.4%
Takada N (1994)	11	85	0.129	[0.066; 0.220]	1.4%
Miyamoto K (1992)	130	792	0.164	[0.139; 0.192]	1.4%
Zhag QN (1991)	42	150	0.280	[0.210; 0.359]	1.4%
Yu PF (2016)	18	42	0.429	[0.277; 0.590]	1.3%
Ye NN (2018)	0	53	0.000	[0.000; 0.067]	1.3%
Chen C (2015)	3	8	0.375	[0.085; 0.755]	0.9%
Pan YP (2017)	5	25	0.200	[0.068; 0.407]	1.2%
Du Y (1992)	215	846	0.254	[0.225; 0.285]	1.4%
Huang HM (2006)	36	100	0.360	[0.266; 0.462]	1.4%
Gao LY (2007)	631	1799	0.351	[0.329; 0.373]	1.4%
Han H (2015)	11	70	0.157	[0.081; 0.264]	1.4%
Yang Y (2018)	0	125	0.000	[0.000; 0.029]	1.4%
Tang F (2018)	85	293	0.290	[0.239; 0.346]	1.4%
Wang W (2013)	0	58	0.000	[0.000; 0.062]	1.3%
Yang J (2016)	0	84	0.000	[0.000; 0.043]	1.4%
Wang CS (2009)	453	1349	0.336	[0.311; 0.362]	1.4%
Wang Q(2021)	13	42	0.310	[0.176; 0.471]	1.3%
Seto J(2020)	1	9	0.111	[0.003; 0.482]	1.0%
Rar V(2019)	252	668	0.377	[0.340; 0.415]	1.4%
Pukhovskaya NM(2019)	582	1599	0.364	[0.340; 0.388]	1.4%
Gao Y(2021)	7	264	0.027	[0.011; 0.054]	1.4%
Jiang BG(2018)	19	627	0.030	[0.018; 0.047]	1.4%
Liu L(2015)	0	113	0.000	[0.000; 0.032]	1.4%
Takano A(2014)	71	3532	0.020	[0.016; 0.025]	1.4%
Khasnatinov MA(2016)	90	445	0.202	[0.166; 0.243]	1.4%
Sato Y(1996)	225	846	0.266	[0.236; 0.297]	1.4%
Random effects model		17513	0.186	[0.131; 0.247]	49.2%
Heterogeneity: I^2 = 99%, τ^2 = 0.0500, p = 0					
**species = I. ricinus**					
Sen E (2011)	10	24	0.417	[0.221; 0.634]	1.2%
Orkun O(2020)	3	30	0.100	[0.021; 0.265]	1.2%
Güner ES(2003)	12	299	0.040	[0.021; 0.069]	1.4%
Karasartova D(2017)	1	4	0.250	[0.006; 0.806]	0.7%
Sakakibara K(2016)	1	248	0.004	[0.000; 0.022]	1.4%
Orkun O (2014)	0	2	0.000	[0.000; 0.842]	0.4%
Random effects model		607	0.087	[0.018; 0.201]	6.4%
Heterogeneity: I^2 = 89%, τ^2 = 0.0278, p < 0.01					
**species = I. sinensis**					
Hou J (2014)	1	94	0.011	[0.000; 0.058]	1.4%
Zheng WQ (2018)	0	4	0.000	[0.000; 0.602]	0.7%
Random effects model		98	0.010	[0.000; 0.039]	2.0%
Heterogeneity: I^2 = 0%, τ^2 = 0, p = 0.69					
**species = I. turdus**					
Kuo CC (2017)	2	5	0.400	[0.053; 0.853]	0.8%
Seo HJ(2021)	14	230	0.061	[0.034; 0.100]	1.4%
Nakayama S(2020)	1	3	0.333	[0.008; 0.906]	0.6%
Random effects model		238	0.183	[0.014; 0.483]	2.7%
Heterogeneity: I^2 = 62%, τ^2 = 0.0517, p = 0.07					
**species = I. vespertlionis**					
Chu CY(2008)	0	4	0.000	[0.000; 0.602]	0.7%
Random effects model		4	0.000	[0.000; 0.221]	0.7%
Heterogeneity: not applicable					
**species = unidentified**					
Han H (2016)	0	72	0.000	[0.000; 0.050]	1.4%
Kim SY(2020)	135	440	0.307	[0.264; 0.352]	1.4%
Seto J(2020)	0	4	0.000	[0.000; 0.602]	0.7%
Rar V(2019)	490	1154	0.425	[0.396; 0.454]	1.4%
Chu CY(2008)	0	1	0.000	[0.000; 0.975]	0.3%
Random effects model		1671	0.127	[0.018; 0.316]	5.1%
Heterogeneity: I^2 = 97%, τ^2 = 0.0495, p < 0.01					
**Random effects model**		**25758**	**0.145**	**[0.108; 0.186]**	**100.0%**
Heterogeneity: I^2 = 99%, τ^2 = 0.0551, p = 0					
Residual heterogeneity: I^2 = 99%, p = 0					

0 0.2 0.4 0.6 0.8

Study	Events	Total	Proportion	95%−CI	Weight
**species = R(B). curtice**					
Niu QL (2010)	59	185	0.319	[0.252; 0.391]	5.3%
**Random effects model**		**185**	**0.319**	**[0.254; 0.388]**	**5.3%**
Heterogeneity: not applicable					
**species = R(B). microplus**					
Zheng WQ (2018)	0	77	0.000	[0.000; 0.047]	5.0%
Han H (2016)	3	86	0.035	[0.007; 0.099]	5.1%
Ghafar A(2020)	1	17	0.059	[0.001; 0.287]	4.0%
Chen Z(2014)	0	10	0.000	[0.000; 0.308]	3.4%
Chu CY(2008)	3	31	0.097	[0.020; 0.258]	4.6%
Hagen RM(2018)	2	148	0.014	[0.002; 0.048]	5.2%
Hou J (2014)	1	20	0.050	[0.001; 0.249]	4.2%
Yu PF (2016)	18	213	0.085	[0.051; 0.130]	5.3%
Mo YM (1994)	0	40	0.000	[0.000; 0.088]	4.7%
Duan CJ (2019)	3	724	0.004	[0.001; 0.012]	5.4%
**Random effects model**		**1366**	**0.021**	**[0.004; 0.050]**	**46.9%**
Heterogeneity: I^2 = 82%, τ^2 = 0.0113, p < 0.01					
**species = R. bursa**					
Orkun O (2014)	0	3	0.000	[0.000; 0.708]	1.8%
Karasartova D(2017)	0	3	0.000	[0.000; 0.708]	1.8%
**Random effects model**		**6**	**0.000**	**[0.000; 0.152]**	**3.7%**
Heterogeneity: I^2 = 0%, τ^2 = 0, p = 1.00					
**species = R. haemaphysaloides**					
Hou J (2014)	0	20	0.000	[0.000; 0.168]	4.2%
Kuo CC (2017)	0	1	0.000	[0.000; 0.975]	0.8%
Zheng WQ (2018)	0	3	0.000	[0.000; 0.708]	1.8%
**Random effects model**		**24**	**0.000**	**[0.000; 0.039]**	**6.8%**
Heterogeneity: I^2 = 0%, τ^2 = 0, p = 1.00					
**species = R. pumilio**					
Sun X (2013)	15	138	0.109	[0.062; 0.173]	5.2%
**Random effects model**		**138**	**0.109**	**[0.062; 0.166]**	**5.2%**
Heterogeneity: not applicable					
**species = R. sanguineus**					
Yu PF (2016)	38	186	0.204	[0.149; 0.269]	5.3%
Orkun O (2014)	0	3	0.000	[0.000; 0.708]	1.8%
Zheng WQ (2018)	0	9	0.000	[0.000; 0.336]	3.3%
**Random effects model**		**198**	**0.041**	**[0.000; 0.309]**	**10.4%**
Heterogeneity: I^2 = 80%, τ^2 = 0.0868, p < 0.01					
**species = R. spp.**					
Niu QL (2010)	14	45	0.311	[0.182; 0.466]	4.8%
Ghafar A(2020)	0	1	0.000	[0.000; 0.975]	0.8%
**Random effects model**		**46**	**0.232**	**[0.008; 0.631]**	**5.6%**
Heterogeneity: I^2 = 27%, τ^2 = 0.0473, p = 0.24					
**species = R. turanicus**					
Orkun O (2014)	0	28	0.000	[0.000; 0.123]	4.5%
Sun X (2013)	0	14	0.000	[0.000; 0.232]	3.8%
Orkun O(2020)	0	9	0.000	[0.000; 0.336]	3.3%
Karasartova D(2017)	0	34	0.000	[0.000; 0.103]	4.6%
**Random effects model**		**85**	**0.000**	**[0.000; 0.011]**	**16.2%**
Heterogeneity: I^2 = 0%, τ^2 = 0, p = 1.00					
**Random effects model**		**2048**	**0.029**	**[0.006; 0.068]**	**100.0%**
Heterogeneity: I^2 = 92%, τ^2 = 0.0428, p < 0.01					
Residual heterogeneity: I^2 = 71%, p < 0.01					

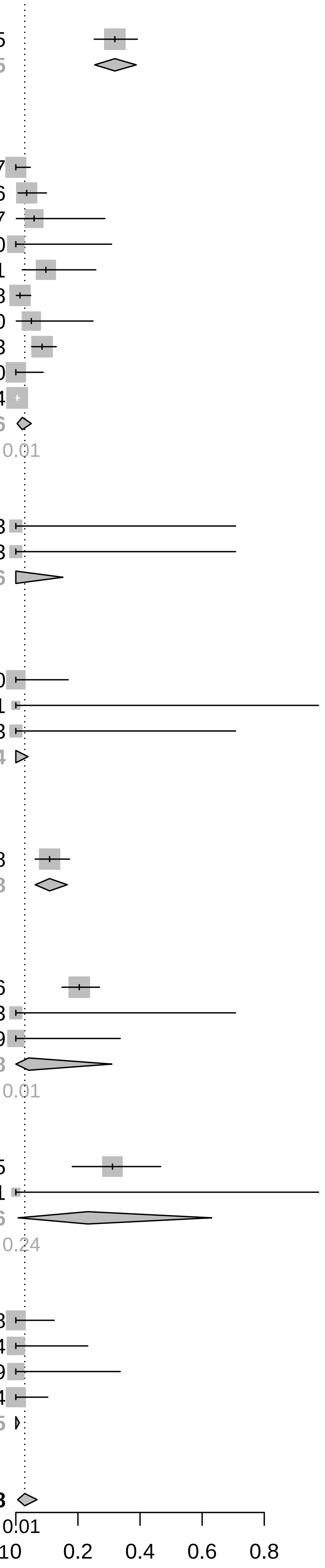

Supplementary Material 6: Funnel plot

(A) Egg's plot of the Asia prevalence of *B. burgdorferi* s.l.in Ixodidae. (B) Funnel plot of the Asia prevalence of *B. burgdorferi* s.l. in Ixodidae.

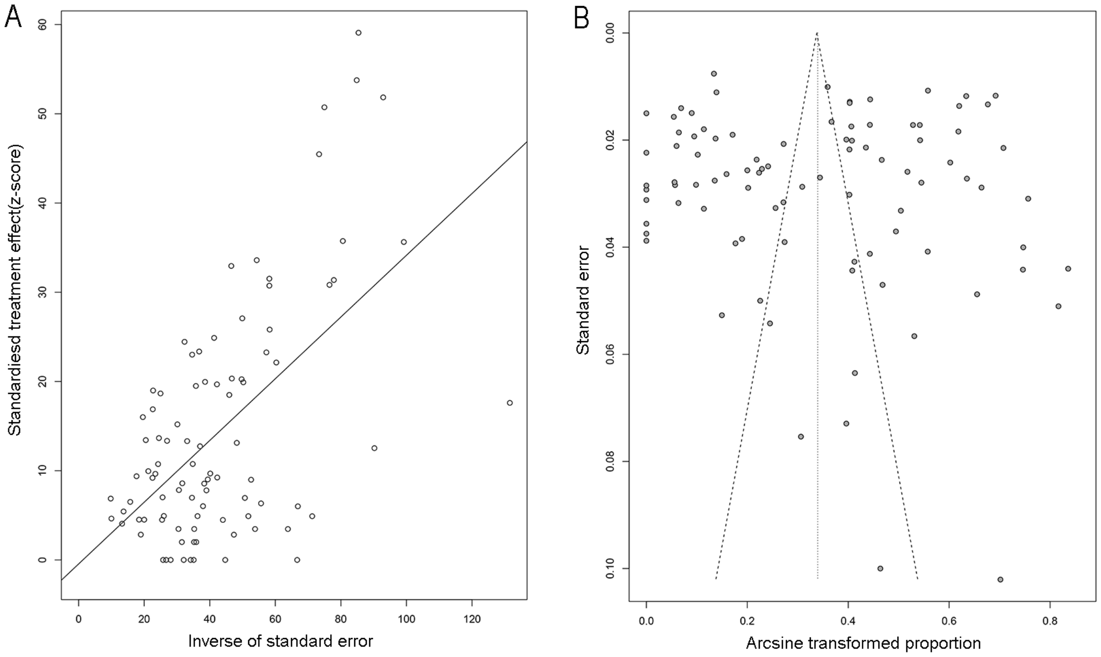

Supplement: Supplementary file 1 [file pathogens-11-00143-s001.zip › pathogens-1494608-supplementary.pdf]
